# Supplementary figures and images for: Novel hormonal therapy versus standard of care—A registry-based comparative effectiveness evaluation for mCRPC-patients
Source: PLoS One. 2024 Feb 14;19(2):e0290833. doi: 10.1371/journal.pone.0290833 (PMC10866493; doi:10.1371/journal.pone.0290833)

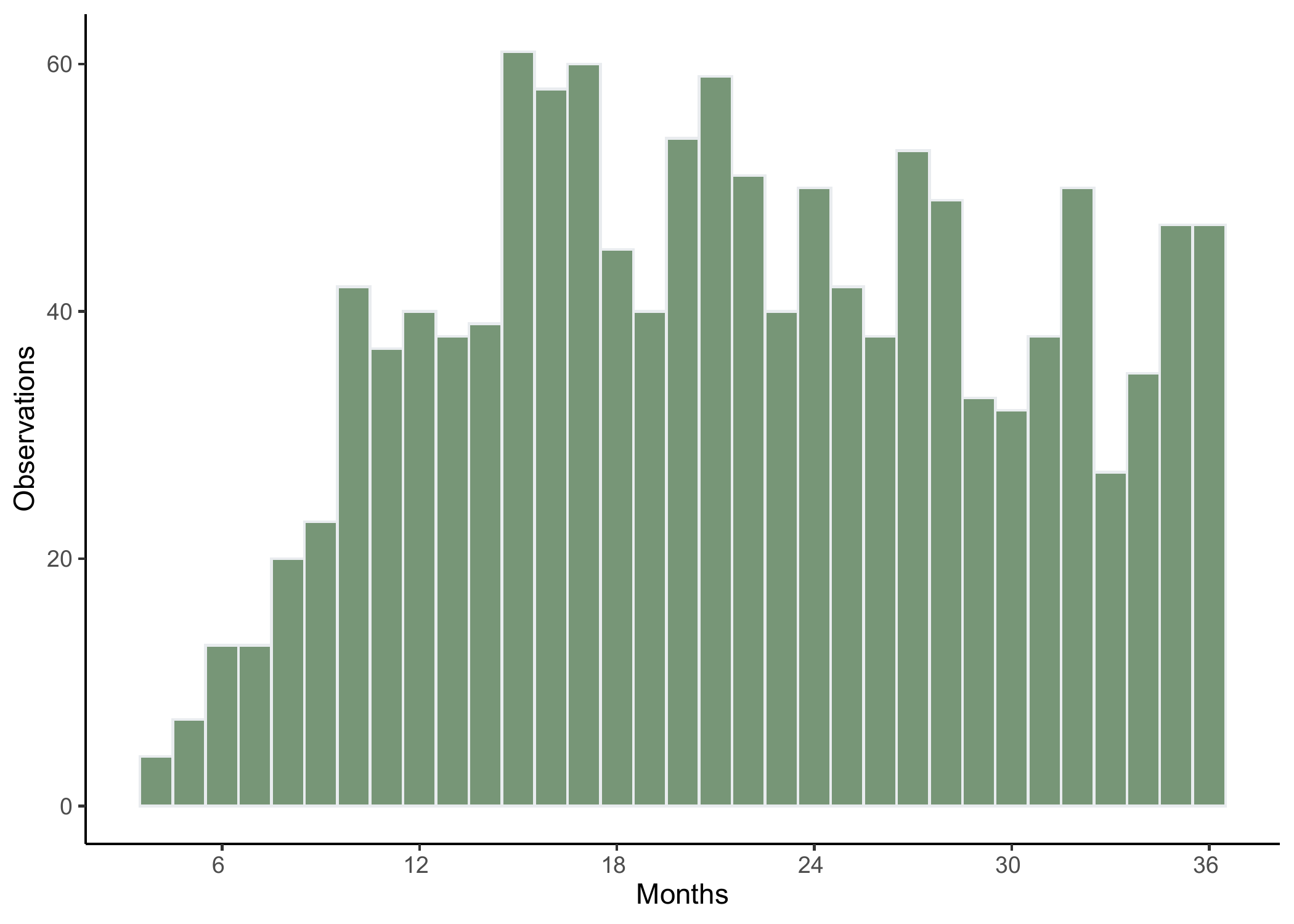

Supplement: S1 Fig — (TIF) [file pone.0290833.s001.tif]

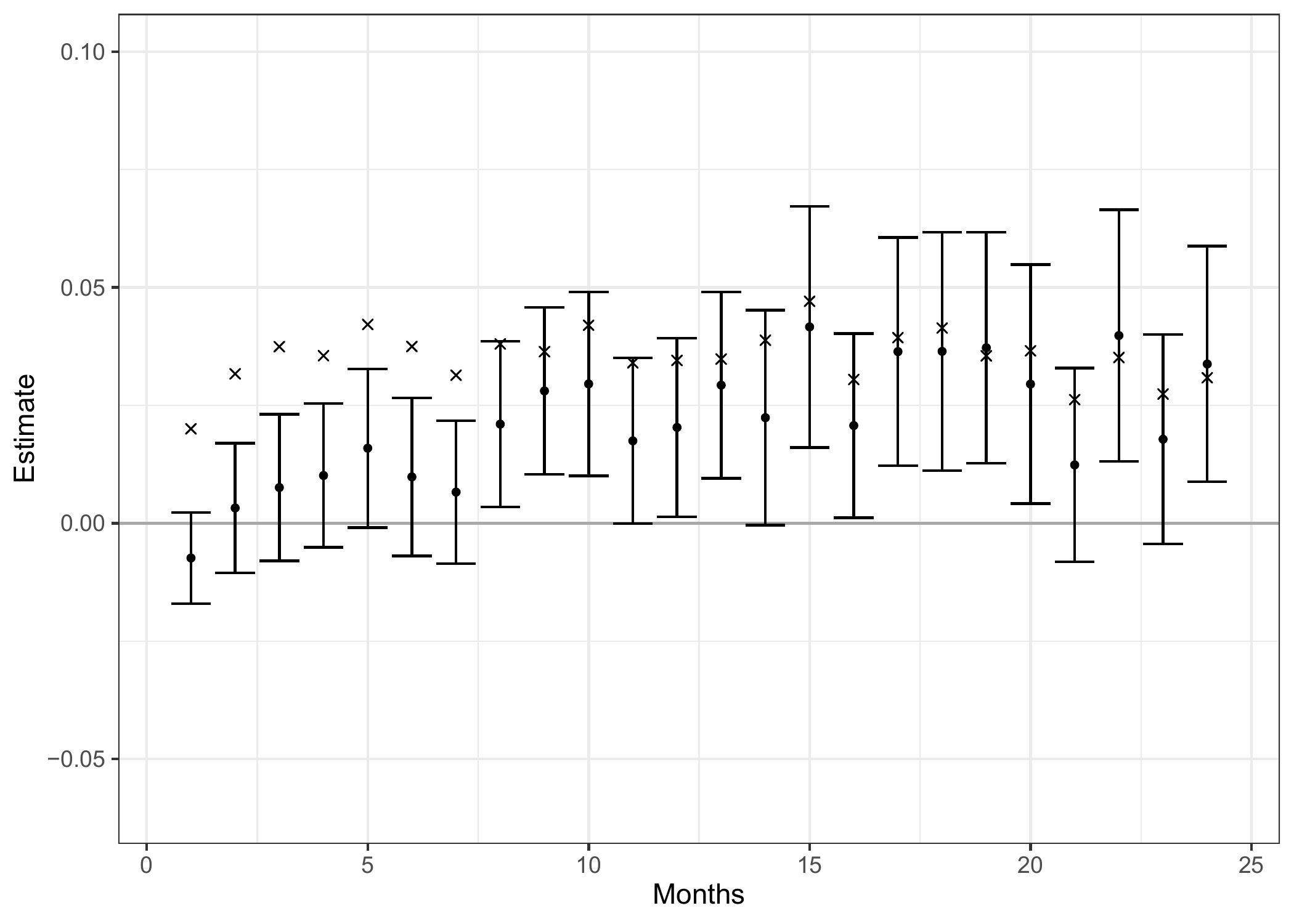

Supplement: S2 Fig — Estimates, 95% Bonferroni confidence intervals and overall mortality for each month (x). Updated entropy weights. (TIF) [file pone.0290833.s002.tif]

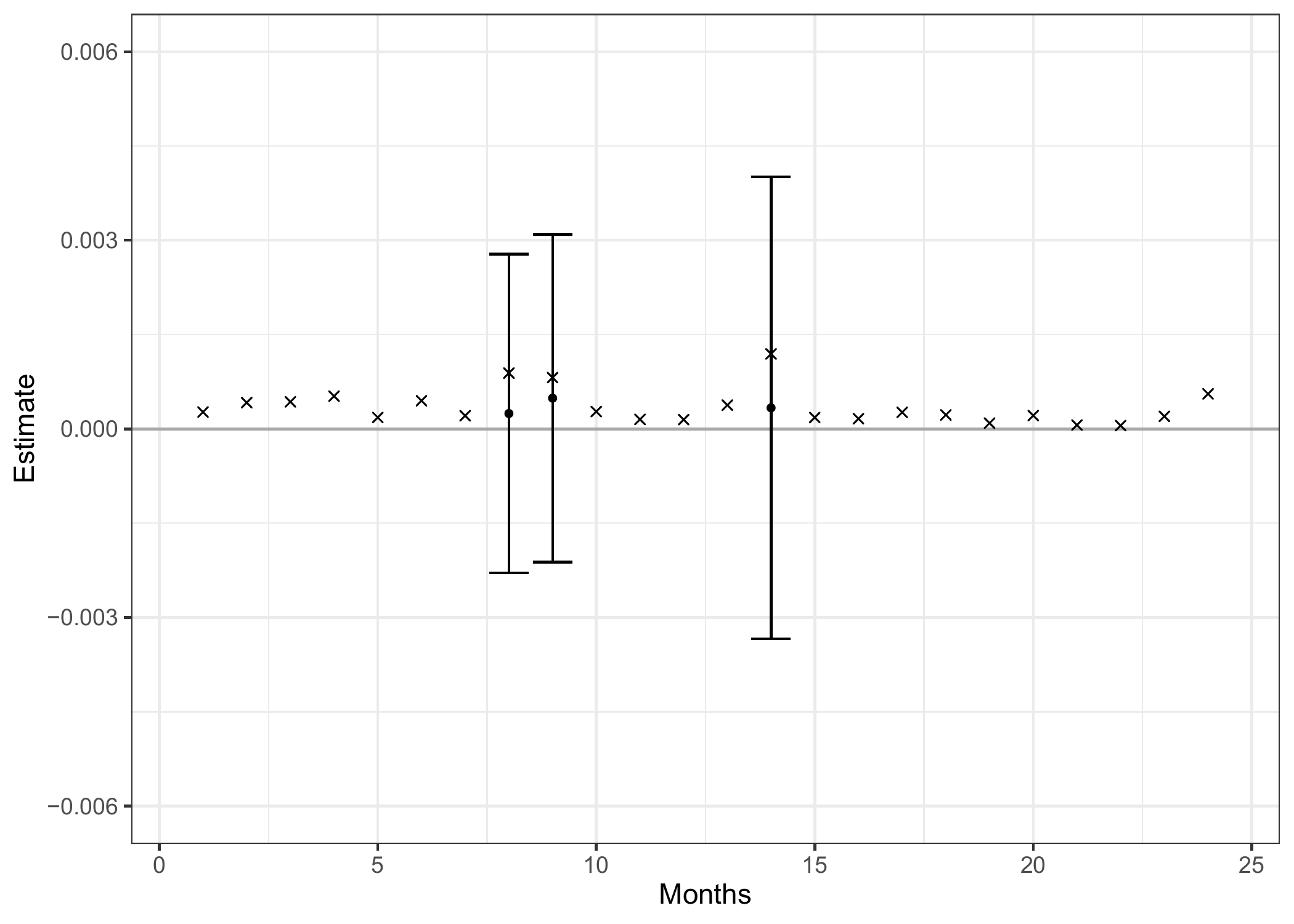

Supplement: S3 Fig — Estimates, 95% Bonferroni confidence intervals and overall level of PAIN for each month (x). The effects are all very small and for the majority of months there are no individuals in the treatment group with prevalence of PAIN. (TIF) [file pone.0290833.s003.tif]

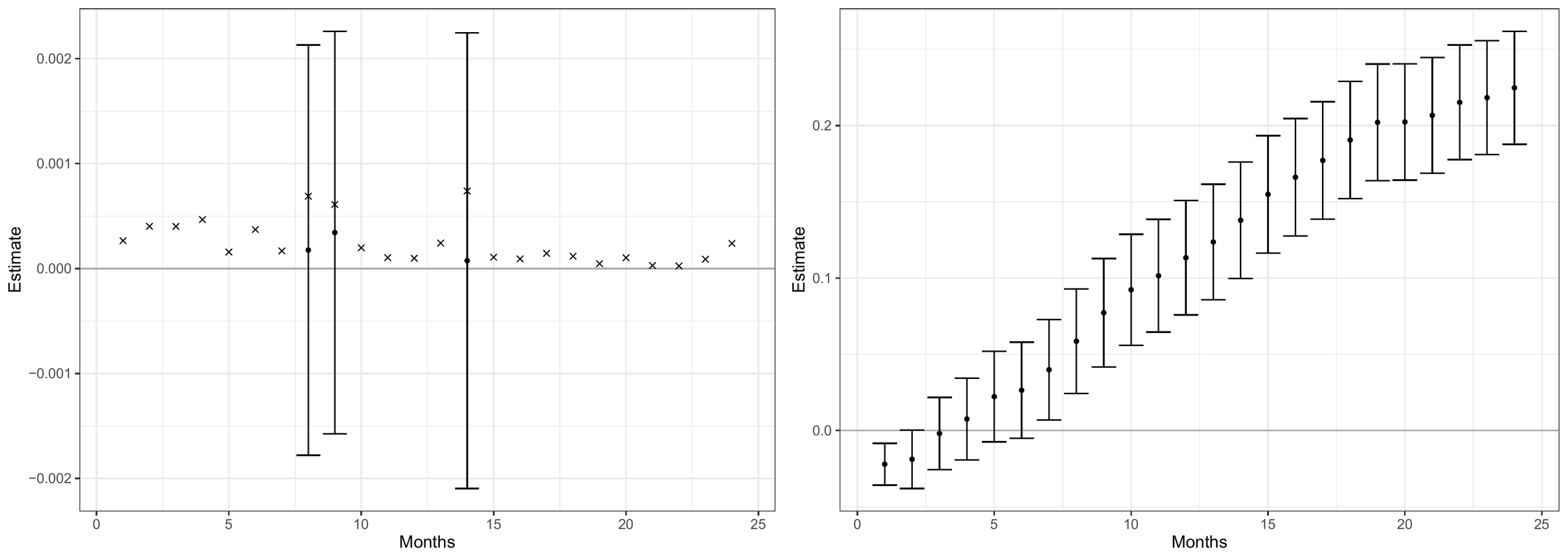

Supplement: S4 Fig — Estimates and 95% Bonferroni corrected confidence intervals. We let all patients who die either have no morbidity outcome or a morbidity outcome (i.e. PAIN = 0 or PAIN = 1). Since the mortality with the NHT is observed to be higher than without a SoC the first case (i.e. PAIN = 0) provides a lower bound estimate of the effectiveness of the NHT while the second one provides an upper bound on PAIN. Lower (left panel) and upper (right panel) bounds of potential effect. (TIF) [file pone.0290833.s004.tif]

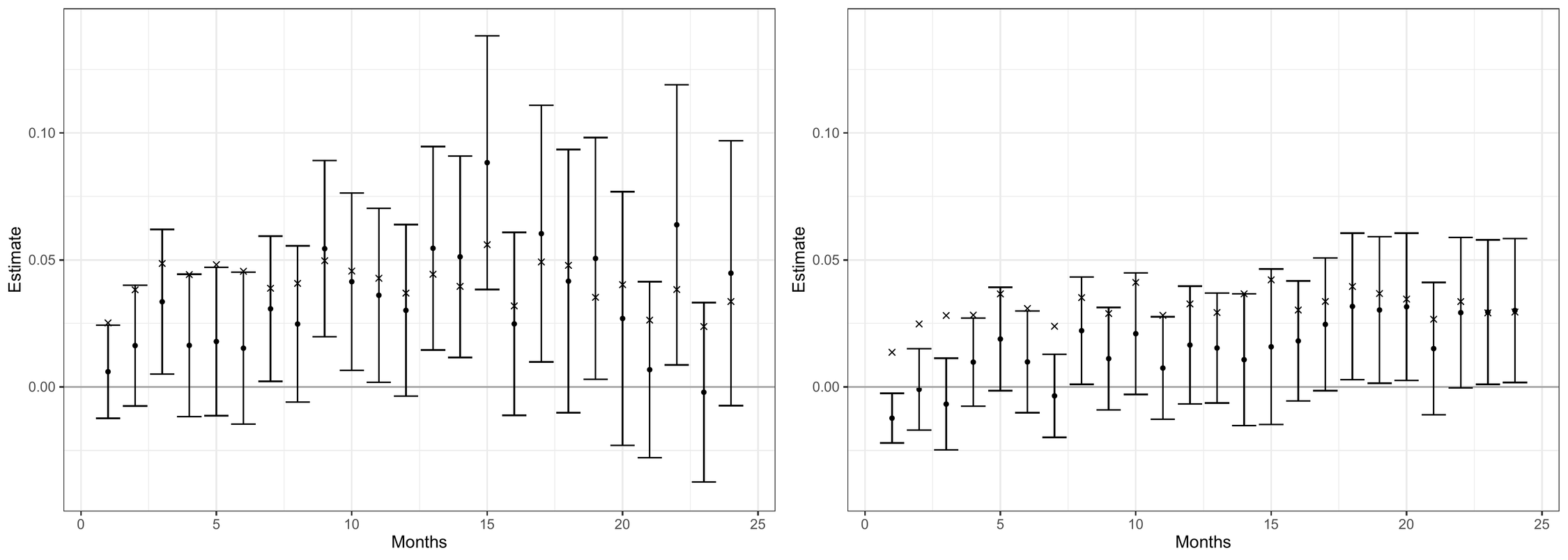

Supplement: S5 Fig — Estimates, 95% Bonferoni corrected confidence intervals and overall level of mortality for each month. Early (left panel) and late (right panel) prescriptions. (TIF) [file pone.0290833.s005.tif]

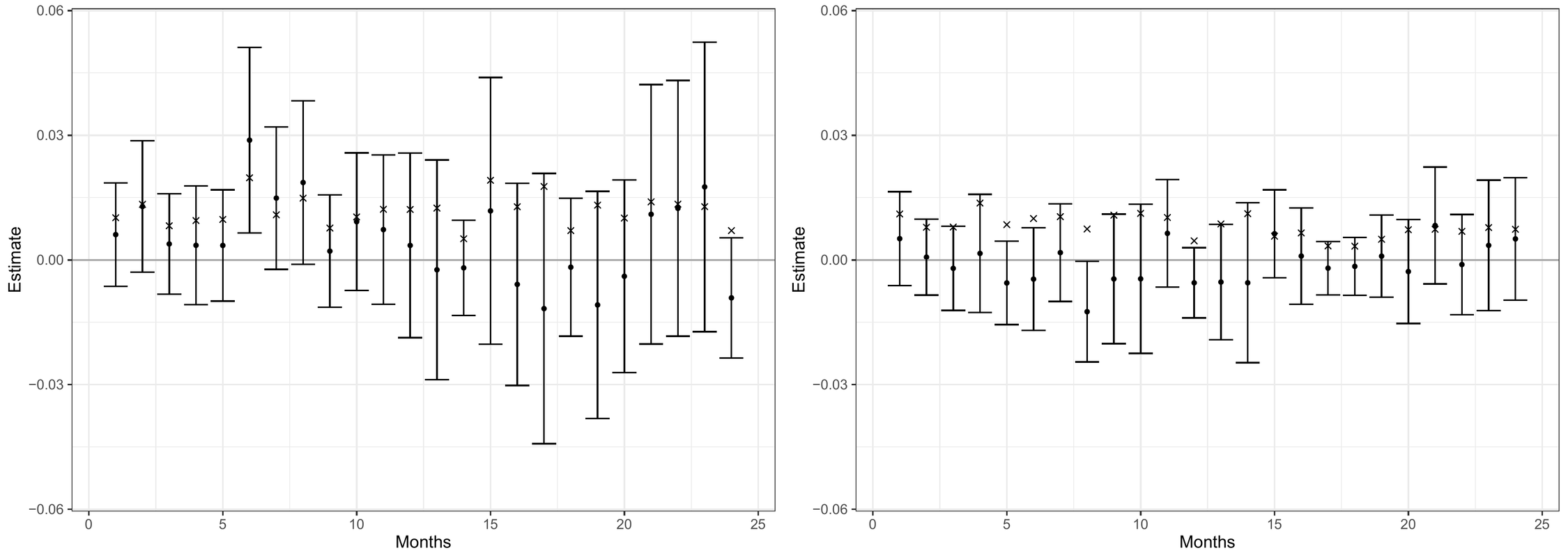

Supplement: S6 Fig — Estimates, 95% Bonferoni corrected confidence intervals and overall level of SRE for each month. Early (left panel) and late (right panel) prescriptions. (TIF) [file pone.0290833.s006.tif]

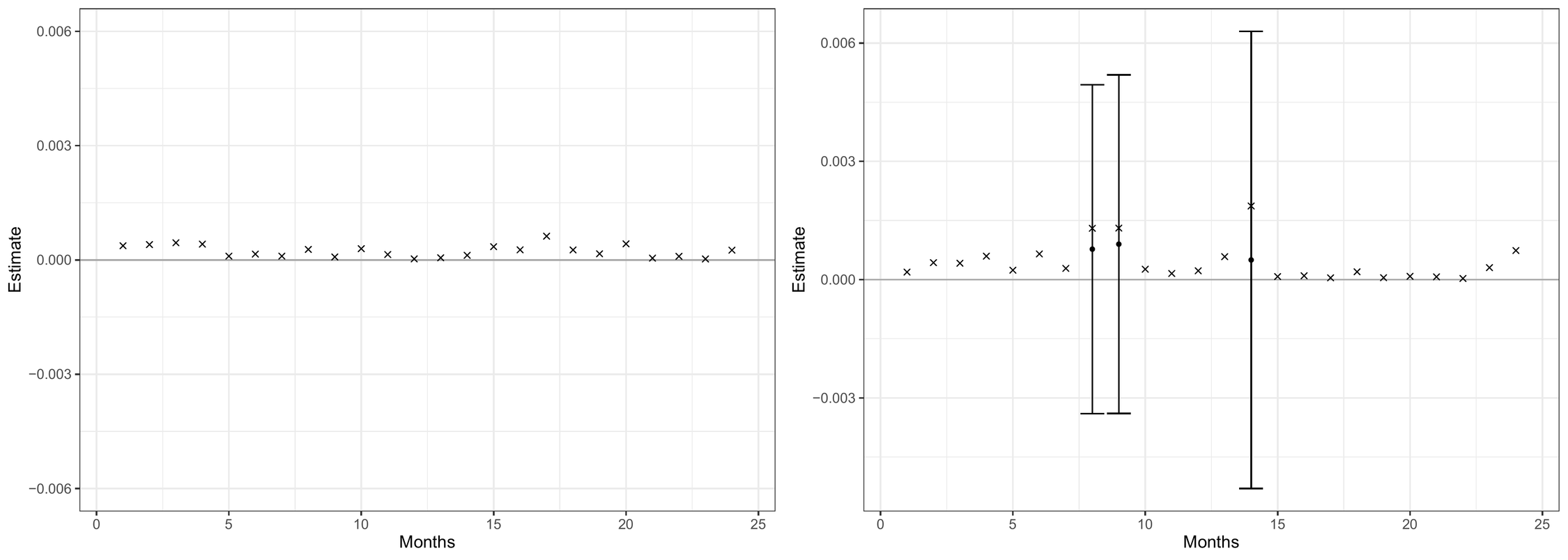

Supplement: S7 Fig — Estimates, 95% Bonferoni corrected confidence intervals and overall level of SRE for each month. Early (left panel) and late (right panel) prescriptions. (TIF) [file pone.0290833.s007.tif]

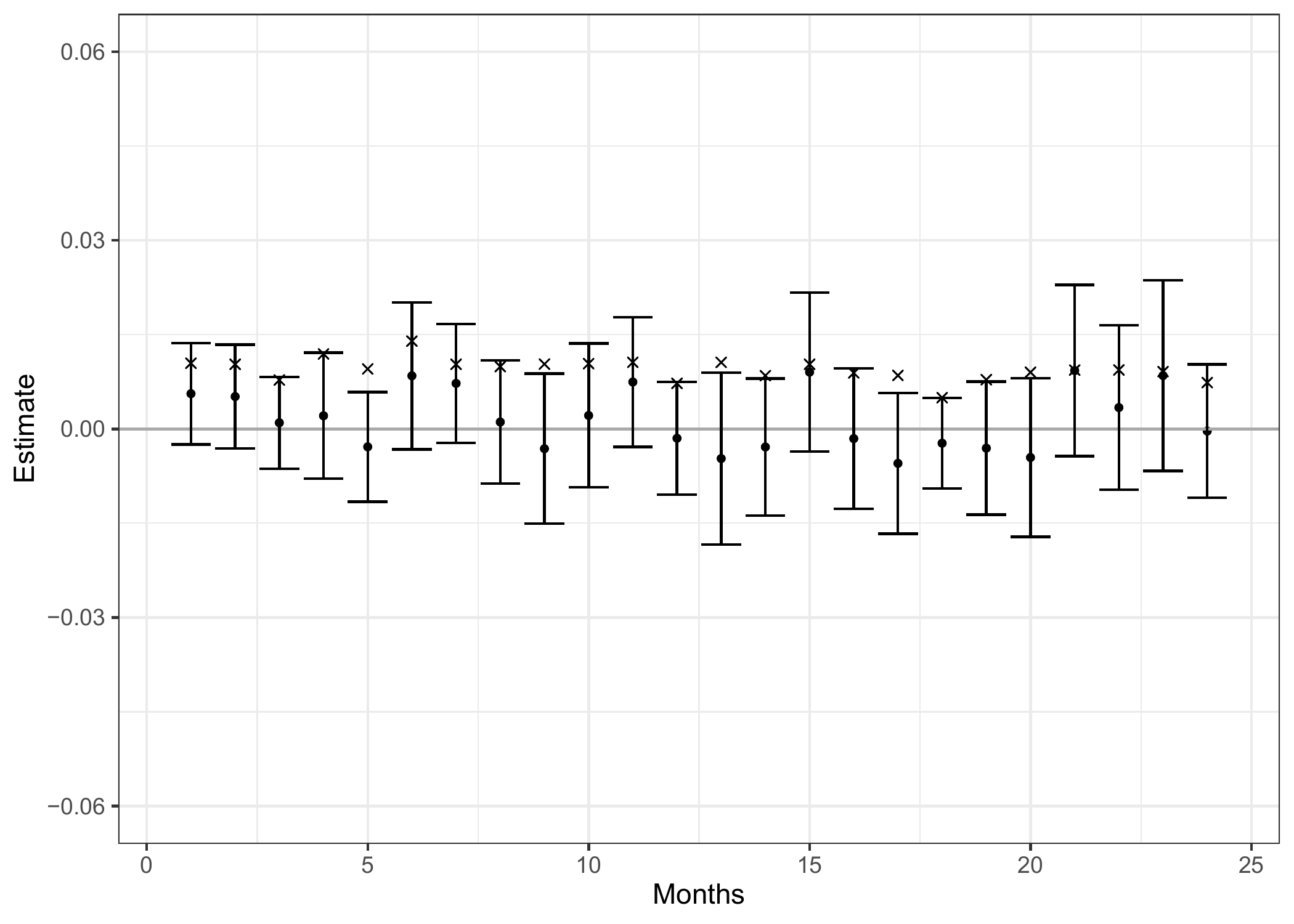

Supplement: S8 Fig — Estimates, 95% Bonferroni confidence intervals and overall mortality for each month. Updated entropy weights. (TIF) [file pone.0290833.s008.tif]

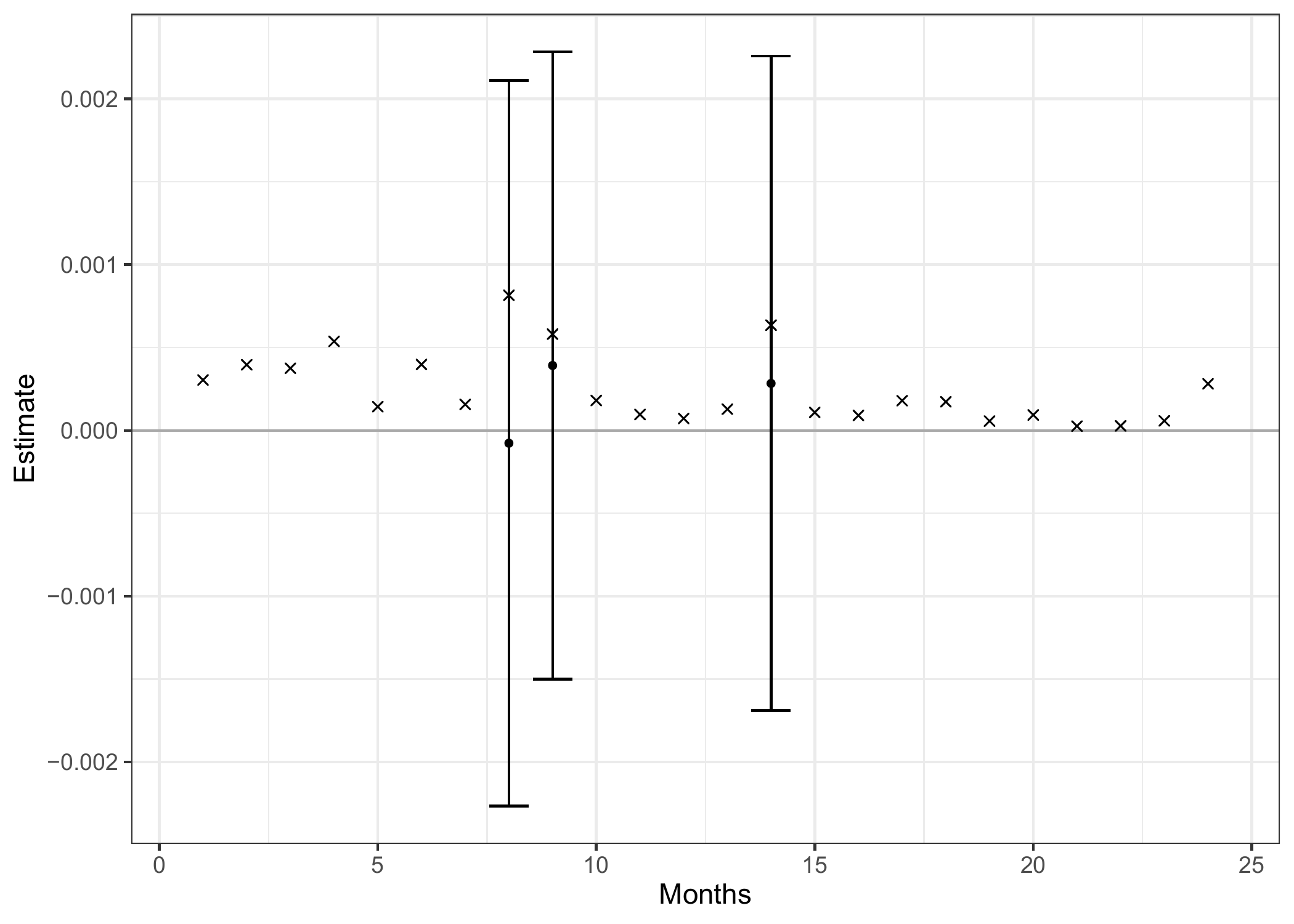

Supplement: S9 Fig — Estimates, 95% Bonferroni confidence intervals and overall mortality for each month. Updated entropy weights. (TIF) [file pone.0290833.s009.tif]

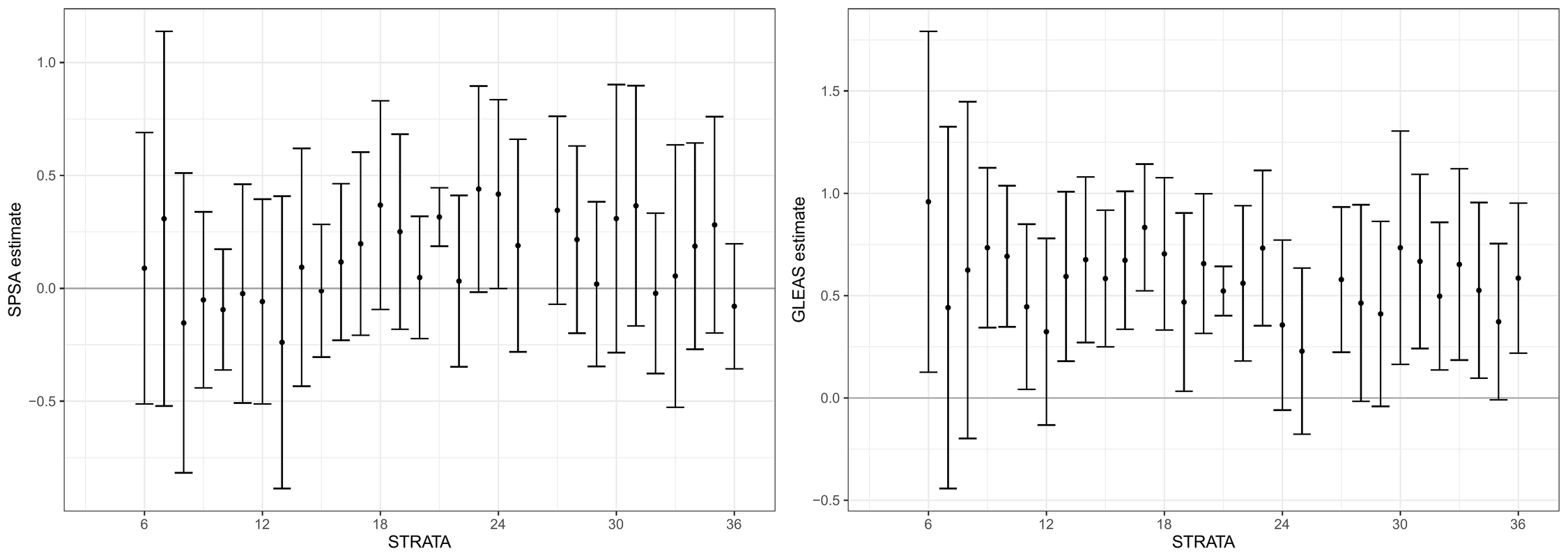

Supplement: S10 Fig — Estimate, 95% Bonferroni corrected confidence and overall level of SPSA and Gleason for each month. SPSA (left) and Gleason (right). Updated entropy weights. (TIF) [file pone.0290833.s010.tif]

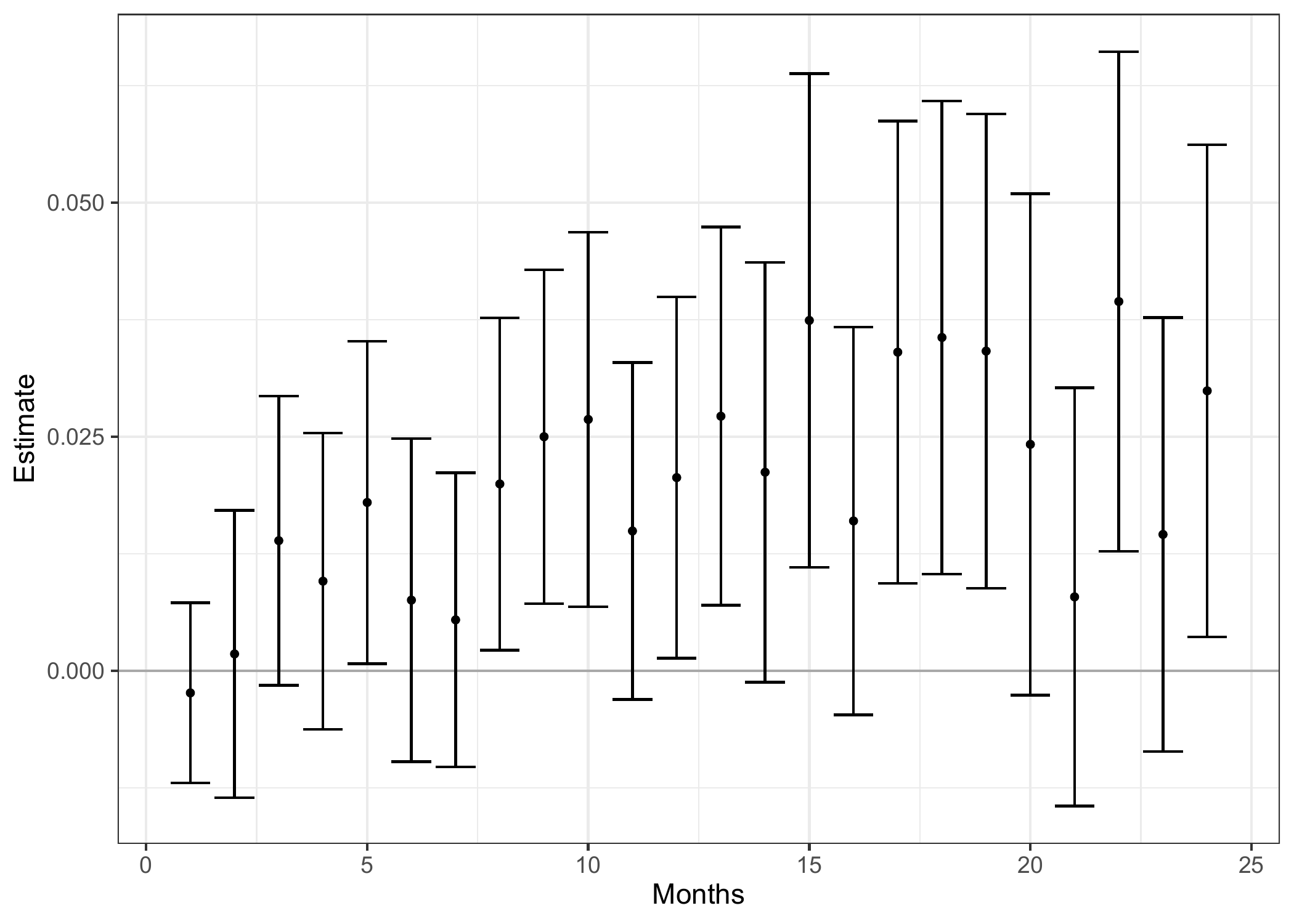

Supplement: S11 Fig — Estimates, 95% Bonferroni confidence intervals and overall mortality for each month. Entropy weights from including GleasSa. (TIF) [file pone.0290833.s011.tif]
