# Supplementary material for: Novel hormonal therapy versus standard of care—A registry-based comparative effectiveness evaluation for mCRPC-patients
Source: PLoS One. 2024 Feb 14;19(2):e0290833. doi: 10.1371/journal.pone.0290833 (PMC10866493; doi:10.1371/journal.pone.0290833)
Supplement: S1 Table — (DOCX) [file pone.0290833.s012.docx]

S1 Table. Result from a discrete time Cox regression.

|  | Coefficient | se | p-value |
| --- | --- | --- | --- |
| TREAT1 | 0.71 | 0.049 | 0.0000 |
| STRATA5 | -0.80 | 0.508 | 0.1147 |
| STRATA6 | -0.31 | 0.447 | 0.4941 |
| STRATA7 | -0.02 | 0.439 | 0.9577 |
| STRATA8 | -0.15 | 0.422 | 0.7276 |
| STRATA9 | -0.16 | 0.418 | 0.7085 |
| STRATA10 | -0.27 | 0.403 | 0.5055 |
| STRATA11 | -0.17 | 0.407 | 0.6675 |
| STRATA12 | -0.26 | 0.405 | 0.5229 |
| STRATA13 | -0.04 | 0.406 | 0.9295 |
| STRATA14 | -0.37 | 0.406 | 0.3614 |
| STRATA15 | -0.34 | 0.399 | 0.3987 |
| STRATA16 | -0.34 | 0.400 | 0.3946 |
| STRATA17 | -0.51 | 0.400 | 0.2059 |
| STRATA18 | -0.53 | 0.405 | 0.1937 |
| STRATA19 | -0.49 | 0.407 | 0.2253 |
| STRATA20 | -0.64 | 0.402 | 0.1089 |
| STRATA21 | -0.68 | 0.401 | 0.0914 |
| STRATA22 | -0.36 | 0.402 | 0.3720 |
| STRATA23 | -0.53 | 0.408 | 0.1920 |
| STRATA24 | -0.44 | 0.402 | 0.2733 |
| STRATA25 | -0.40 | 0.406 | 0.3267 |
| STRATA26 | -0.59 | 0.409 | 0.1516 |
| STRATA27 | -0.50 | 0.402 | 0.2143 |
| STRATA28 | -0.72 | 0.405 | 0.0744 |
| STRATA29 | -0.58 | 0.413 | 0.1607 |
| STRATA30 | -0.51 | 0.413 | 0.2208 |
| STRATA31 | -0.76 | 0.411 | 0.0654 |
| STRATA32 | -0.63 | 0.404 | 0.1172 |
| STRATA33 | -0.48 | 0.418 | 0.2502 |
| STRATA34 | -0.39 | 0.410 | 0.3379 |
| STRATA35 | -0.71 | 0.406 | 0.0787 |
| STRATA36 | -0.68 | 0.405 | 0.0947 |
