# Supplementary material for: Novel hormonal therapy versus standard of care—A registry-based comparative effectiveness evaluation for mCRPC-patients
Source: PLoS One. 2024 Feb 14;19(2):e0290833. doi: 10.1371/journal.pone.0290833 (PMC10866493; doi:10.1371/journal.pone.0290833)
Supplement: S2 Table — Means (standard deviations). (DOCX) [file pone.0290833.s013.docx]

S2 Table. Summary statistics

|  | Age | Number of months with skeleton metastases |
| --- | --- | --- |
| SoC | 69.17 (9.02) | 0.84 (3.53) |
| NHT | 70.71 (8.19) | 8.96 (7.75) |
| Total | 69.17 (9.02) | 0.85 (3.57) |

Means (standard deviations).
