# Supplementary material for: Novel hormonal therapy versus standard of care—A registry-based comparative effectiveness evaluation for mCRPC-patients
Source: PLoS One. 2024 Feb 14;19(2):e0290833. doi: 10.1371/journal.pone.0290833 (PMC10866493; doi:10.1371/journal.pone.0290833)
Supplement: S1 Text — (DOCX) [file pone.0290833.s014.docx]

**S1 Text. Analysis of missing in the proxy variables**

Figure A displays the fraction missing for the NHT patients and the weighted average fraction missing for the SoC patients at each month of treatment. The figure clearly shows that the fraction missing for SPSA (left panel) and GleasSa (mid panel) are fairly balanced between the two groups. However the fraction missing for the Mstad (right panel) is substantially higher (around 40 %) for the SoC than for the NHT patients (around zero). Given this large difference in missingness, Mstad is not a valid proxy outcome to be used in the assessment.


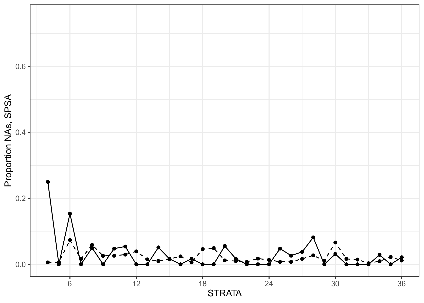

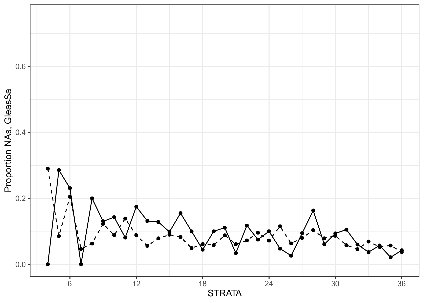

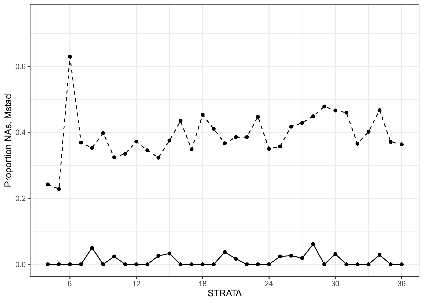


**Figure A. Fraction missing.** SPSA (left), GleasSa (middle) and Mstad (right), - - SoC and --- NHT.

As a means to test for missing at random, we regress the indicator variable of a missing value on the covariates used in the main analysis and the treatment indicator *T*. Given the included covariates, the level of missing data do not differ between the NHT and SoC patients except in stratum 4, 5 and 26 for GleasSa. The results displayed in Figure B show that there are less missing on this variable for NHT in stratum 4 and 26, and more missing in stratum 5. Thus, with the exception for these three strata we can treated the missing observations as missing at random given the covariates.

As the degree of missing observations is low in general we simply remove the patients with missing from the sensitivity analyses of remaining stratum.


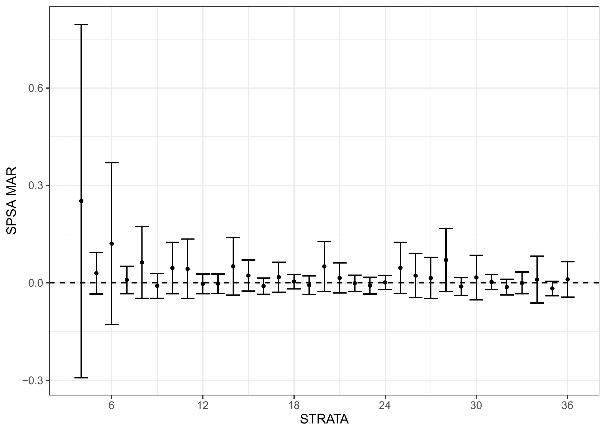

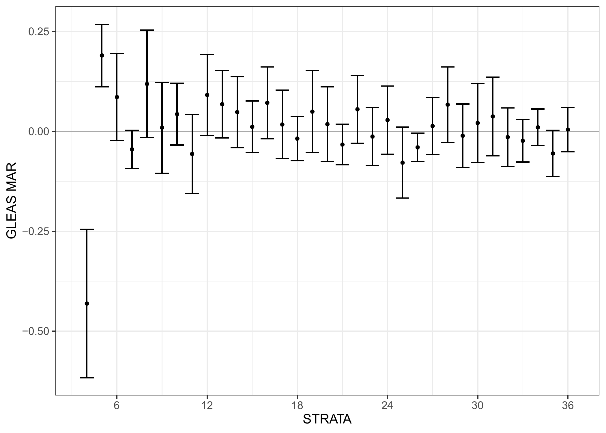


**Figure B. Missing at random, WLS estimates and 95% Bonferroni corrected confidence intervals.** SPSA (left) and GleasSa (right).
