# Supplementary material for: Novel hormonal therapy versus standard of care—A registry-based comparative effectiveness evaluation for mCRPC-patients
Source: PLoS One. 2024 Feb 14;19(2):e0290833. doi: 10.1371/journal.pone.0290833 (PMC10866493; doi:10.1371/journal.pone.0290833)
Supplement: S2 Text — (DOCX) [file pone.0290833.s015.docx]

**S2 Text. Inference under confounding**

The main analysis was conducted using the following regression model on the three outcomes $Y_{im},$ separately for each month *m* (for simplicity, without the subscript $m=1,\ldots,24$) and where the month of treatment after diagnosis is $w=4,\ldots,36$,

$$Y_{i}=\alpha_{w}+\beta T_{i}+\varepsilon_{i}, i=1,\ldots,20,757, (Eq.1)$$

If there is an unobservable *u,* not included in the entropy balancing, the regression model can be formulated

$Y_{i}={\alpha'}_{w}+\beta^{'}T_{i}+\delta u_{i}+\epsilon_{i} i=1,\ldots,20,757 (Eq.2)$.

If $\gamma_{w}\neq0$ in the linear projection of *u* on *T*,

$$u_{i}=\pi_{w}+\gamma_{w}T_{i}+\eta_{i}, i=1,\ldots,20,757 (Eq.3)$$

then *u* is a confounder. Thus, based on the additive separable assumption in Eq 2 it is easy to show that $\hat{\beta}$in Eq. 1 converge to $(\beta+\gamma_{w}\times\delta)$.

We want to answer the question of under a given value of $\gamma_{w},$ what association $\delta$ is the smallest possible to render a negative and statistically significant estimate of treatment on the outcome.

We can answer this question by using the fact that $\hat{\beta}$is asymptotically normal distributed, centered around $(\beta+\gamma_{w}\times\delta)$ and that the standard error under the alternative of confounding is equal to $\hat{s}$, the originally estimated standard errors. We examine the upper limit of the confidence interval

$\hat{\beta}+z_{\alpha/2}\hat{s}=\beta+z_{\alpha/2}\hat{s}+\gamma_{w}\times\delta$,

and want to find the smallest possible value of$\delta$ for which $\beta+z_{\alpha/2}\hat{s}\leq0$, i.e. where we have a negative and statistically significant effect. This is found by solving for $\delta$ in the equation above, at the point where $\beta+z_{\alpha/2}\hat{s}=0.$ We denote this value $\delta^{l}=(\hat{\beta}+z_{\alpha/2}\hat{s})$/$\gamma_{w}$, being the worst case for the least upper bound of the association necessary, for the estimates on mortality to be negative and statistically significant.

We use placebo regressions,

$$Z_{i}=\pi_{w}+\gamma_{w}T_{i}+\eta_{i}, i=1,\ldots,20,757 (Eq.4)$$

where $Z_{i}$ is the proxy outcome, i.e. SPSA or The Gleason score (GleasSa). GleasSa was seen not to be balanced between the two groups. It is thus a confounder if it is also associated with the outcome, that is, $\delta\neq0$in the setup above. The maximum estimated $\gamma_{w}$for GleasSa was found at waiting time of 6 months, with the estimate from Eq.4 being: $\hat{\gamma}_{6}$ = 0.96. This estimate is then used to find the smallest possible value of $\delta$ for each month $m=1,\ldots,24,$ to change the inference in the mortality analysis.

The results from this exercise are displayed in column (2) of Table A.

To get an understanding of the magnitude of these correlations, we in addition estimated$\delta$ for each month $m=1,\ldots,24$ on *Months with skeleton metastases (metastases)* and *Age*. As these two variables are included in the entropy balancing, the previous weighted regression cannot be used in the analysis. Instead we estimate the following regression model on the SoC patents

$$Y_{i}=\alpha_{mw}+{X'}_{iw}\beta_{mw}+{\delta_{m}D_{i,w}+\varepsilon}_{i}, m=1,\ldots,24, i=1,\ldots,19,456 (Eq.5)$$

where $D_{i,w}$ is the standardized (zero mean and one standard deviation) variable *Age* or *Metastases*. The $X_{iw}$ are all the covariates used in the entropy balancing schemes except for age in the age regression and metastases in the metastases regression. These estimates are presented in columns (3) and (4) of Table A. In addition, we also present a column (5) with the values of $\hat{\delta}_{m}$ from Eq. 5 with $D_{i,w}$ being the standardized Gleason score.

For all comparisons, the lower limit is substantially larger than the estimates.

**Table A. Results from the sensitivity analyses**

| Month | Lower limit ($\hat{\delta}_{m}^{l})$ | | Metastases | Age | GleasSa |
| --- | --- | --- | --- | --- | --- |
| 1 | 0.0054 | 0.00079 (0.0013) | | 0.00031 (0.00042) | 0.00082 (0.00017) |
| 2 | 0.0210 | 0.00205 (0.0013) | | 0.00034 (0.00037) | 0.00110 (0.00016) |
| 3 | 0.0273 | 0.00166 (0.0012) | | 0.00144 (0.00040) | 0.00131 (0.00015) |
| 4 | 0.0292 | 0.00225 (0.0013) | | 0.00188 (0.00040) | 0.00146 (0.00015) |
| 5 | 0.0369 | 0.00212 (0.0013) | | 0.00123 (0.00039) | 0.00164 (0.00015) |
| 6 | 0.0304 | 0.00279 (0.0013) | | 0.00183 (0.00037) | 0.00177 (0.00016) |
| 7 | 0.0265 | 0.00225 (0.0013) | | 0.00163 (0.00037) | 0.00172 (0.00016) |
| 8 | 0.0427 | 0.00362 (0.0013) | | 0.00201 (0.00039) | 0.00176 (0.00016) |
| 9 | 0.0488 | 0.00336 (0.0013) | | 0.00229 (0.00038) | 0.00184 (0.00016) |
| 10 | 0.0511 | 0.00284 (0.0014) | | 0.00202 (0.00039) | 0.00178 (0.00016) |
| 11 | 0.0381 | 0.00339 (0.0013) | | 0.00205 (0.00039) | 0.00175 (0.00016) |
| 12 | 0.0428 | 0.00226 (0.0012) | | 0.00156 (0.00036) | 0.00184 (0.00016) |
| 13 | 0.0528 | 0.00334 (0.0014) | | 0.00149 (0.00036) | 0.00194 (0.00016) |
| 14 | 0.0500 | 0.00452 (0.0014) | | 0.00248 (0.00041) | 0.00198 (0.00016) |
| 15 | 0.0722 | 0.00380 (0.0014) | | 0.00204 (0.00039) | 0.00196 (0.00016) |
| 16 | 0.0423 | 0.00390 (0.0013) | | 0.00176 (0.00038) | 0.00208 (0.00016) |
| 17 | 0.0650 | 0.00419 (0.0014) | | 0.00158 (0.00038) | 0.00206 (0.00016) |
| 18 | 0.0641 | 0.00461 (0.0014) | | 0.00160 (0.00037) | 0.00207 (0.00016) |
| 19 | 0.0652 | 0.00409 (0.0014) | | 0.00181 (0.00039) | 0.00203 (0.00016) |
| 20 | 0.0581 | 0.00343 (0.0014) | | 0.00230 (0.00040) | 0.00204 (0.00016) |
| 21 | 0.0346 | 0.00326 (0.0014) | | 0.00202 (0.00039) | 0.00195 (0.00016) |
| 22 | 0.0714 | 0.00232 (0.0014) | | 0.00215 (0.00040) | 0.00188 (0.00016) |
| 23 | 0.0430 | 0.00279 (0.0014) | | 0.00184 (0.00038) | 0.00192 (0.00016) |
| 24 | 0.0634 | 0.00304 (0.0014) | | 0.00253 (0.00041) | 0.00185 (0.00016) |

The lower limit ($\hat{\delta}_{m}^{l})$ is the derived lower limit of a potentially reversed effect for each month. Estimates are the estimated association (standard errors in parentheses) between mortality and metastases, age and GleasSa, respectively.
