# Supplementary material for: Novel hormonal therapy versus standard of care—A registry-based comparative effectiveness evaluation for mCRPC-patients
Source: PLoS One. 2024 Feb 14;19(2):e0290833. doi: 10.1371/journal.pone.0290833 (PMC10866493; doi:10.1371/journal.pone.0290833)
Supplement: S3 Text — (DOCX) [file pone.0290833.s016.docx]

**S3 Text. New grading system for Gleason score**

The main changes in 2014 were to (i) assign cribriform glands as Gleason pattern 4, irrespective of size; assign (ii) glomeruloid glands as Gleason pattern 4, irrespective of size; (iii) Grade mucinous carcinoma of the prostate based on its underlying growth pattern rather than grading them all as pattern four and (iv) not to assign a Gleason grade to IDC of the prostate without invasive carcinoma (1). Our interpretation is that the major consequence of the modification is that some of the earlier patients with a Gleason score 3 now would have been classified with a Gleason score 4 and that the higher Gleason score of the NHT patients may be a consequence of the modification of grading.

For this reason, we conducted the same placebo regressions separately for two subgroups of NHT patients. First, we let the treatment group consist only of patients diagnosed before December 2014. Secondly, we did the same analysis for patients diagnosed after this date. The results from these analyses are presented in Figure A.


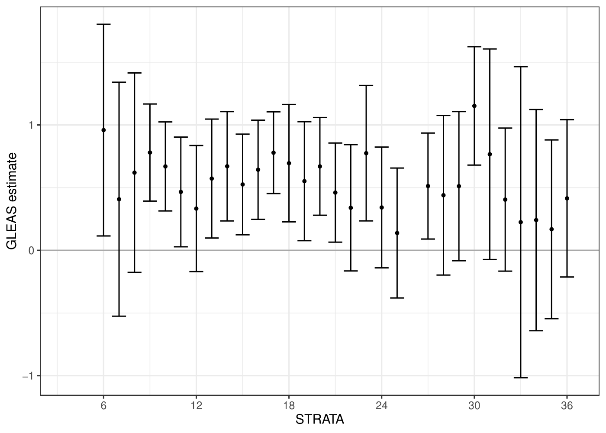

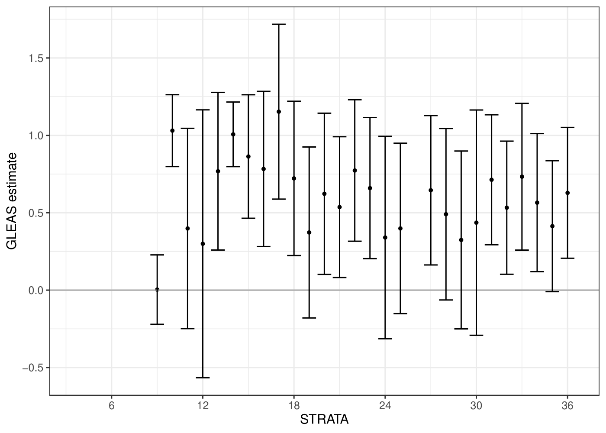


Figure A: Estimates, 95 percent Bonferroni confidence intervals and overall mortality for each month. Treatment group with diagnosis before December 1, 2014 (left) and Treatment group with diagnosis after December 1, 2014 (right)

1. Epstein, J. I., Egevad, L., Amin, M. B., Delahunt, B., Srigley, J. R. and Humphrey, P. A. The 2014 International Society of Urological Pathology (ISUP) consensus conference on Gleason grading of prostatic carcinoma. The American Journal of Surgical Pathology 2016;40(2): 244-252.
