# Supplementary material for: Novel hormonal therapy versus standard of care—A registry-based comparative effectiveness evaluation for mCRPC-patients
Source: PLoS One. 2024 Feb 14;19(2):e0290833. doi: 10.1371/journal.pone.0290833 (PMC10866493; doi:10.1371/journal.pone.0290833)
Supplement: Attachment — Submitted filename: PONE-D-23-09599_reviewer_vetted.pdf [file pone.0290833.s018.pdf]

PLOS ONE

Novel hormonal therapy versus Standard of Care -- a registry-based comparative effectiveness evaluation for mCRPC-patients

--Manuscript Draft--

**Manuscript Number:**	PONE-D-23-09599
**Article Type:**	Research Article
**Full Title:**	Novel hormonal therapy versus Standard of Care -- a registry-based comparative effectiveness evaluation for mCRPC-patients
**Short Title:**	A comparative Effectiveness Evaluation of New Hormonal Therapy versus Standard of Care
**Corresponding Author:**	Sophie Langenskiöld<br>Uppsala University Faculty of Medicine: Uppsala Universitet Medicinska fakulteten<br>Uppsala, Uppsala SWEDEN
**Keywords:**	
**Abstract:**	Background<br>This paper presents results from one of the few comparative effectiveness evaluations of novel antiandrogen medications (NHT) against standard of care (SoC) for patients suffering from metastatic castrate-resistant prostate cancer (mCRPC).<br><br>Methods<br>The design and the analysis are published in a protocol before accessing outcome data. Two groups of patients are balanced on hundreds of important covariates measured before the prostate cancer diagnosis and up to the date of the prescription. While the design yields balance on the observed covariates, one cannot discard the possibility that unobserved confounders are not balanced. The unconfoundedness assumption is assessed by estimating placebo regressions on two health measures, not included in the design but added together with the outcome data after protocol publication.<br><br>Results<br>We find a substantial increase in mortality for patients prescribed with NHT rather than SoC. However, based on the results from one of the two placebo regressions, we cannot rule out that the difference in mortality may be due to confounding. However, using a bounding strategy of the effect, we can rule out that NHT reduces mortality compared to SoC. We can also rule out a positive effect on skeleton-related events (SRE) for the NHT patients.<br><br>Conclusions<br>Generally, the SoC for this group of patients is docetaxel. Given the substantially higher costs of NHT, the finding of no positive effects from NHT on both mortality and SRE is important. More comparative studies, including studies analysing quality of life outcomes, are thus needed.
**Order of Authors:**	Sophie Langenskiöld
**Additional Information:**	
**Question**	**Response**
**Financial Disclosure**<br><br>Enter a financial disclosure statement that describes the sources of funding for the work included in this submission. Review the submission guidelines for detailed requirements. View published research articles from *PLOS ONE* for specific	No, the funders had no role in the study design, data collection and analysis, decision to publish, or preparation of the manuscript.

Powered by Editorial Manager® and ProduXion Manager® from Aries Systems Corporation

examples.<br><br>This statement is required for submission and **will appear in the published article** if the submission is accepted. Please make sure it is accurate.<br><br>**Unfunded studies**<br>Enter: *The author(s) received no specific funding for this work.*<br><br>**Funded studies**<br>Enter a statement with the following details:<br>• Initials of the authors who received each award<br>• Grant numbers awarded to each author<br>• The full name of each funder<br>• URL of each funder website<br>• Did the sponsors or funders play any role in the study design, data collection and analysis, decision to publish, or preparation of the manuscript?<br>• **NO** - Include this sentence at the end of your statement: *The funders had no role in study design, data collection and analysis, decision to publish, or preparation of the manuscript.*<br>• **YES** - Specify the role(s) played.<br><br>* typeset	
**Competing Interests**<br><br>Use the instructions below to enter a competing interest statement for this submission. On behalf of all authors, disclose any competing interests that could be perceived to bias this work—acknowledging all financial support and any other relevant financial or non-financial competing interests.<br><br>This statement is required for submission and **will appear in the published article** if the submission is accepted. Please make sure it is accurate and that any funding sources listed in your Funding Information later in the submission form are also declared in your Financial Disclosure statement.	No,. The authors have declared that no competing interests exist.

View published research articles from *PLOS ONE* for specific examples.<br><br>**NO authors have competing interests**<br><br>Enter: *The authors have declared that no competing interests exist.*<br><br>**Authors with competing interests**<br><br>Enter competing interest details beginning with this statement:<br><br>*I have read the journal's policy and the authors of this manuscript have the following competing interests: [insert competing interests here]*<br><br>* typeset	
**Ethics Statement**<br><br>Enter an ethics statement for this submission. This statement is required if the study involved:<br><br>• Human participants<br>• Human specimens or tissue<br>• Vertebrate animals or cephalopods<br>• Vertebrate embryos or tissues<br>• Field research<br><br>Write "N/A" if the submission does not require an ethics statement.<br><br>General guidance is provided below. Consult the submission guidelines for detailed instructions. **Make sure that all information entered here is included in the Methods section of the manuscript.**	The data analyzed in the study consist of data from Statistics Sweden, National Bureau of Health & Welfare, and National Prostate Cancer Register, and their use has been approved by the regional ethics review board in Sweden (reference number 2017/02823).

Powered by Editorial Manager® and ProduXion Manager® from Aries Systems Corporation

**Format for specific study types**<br><br>**Human Subject Research (involving human participants and/or tissue)**<br>• Give the name of the institutional review board or ethics committee that approved the study<br>• Include the approval number and/or a statement indicating approval of this research<br>• Indicate the form of consent obtained (written/oral) or the reason that consent was not obtained (e.g. the data were analyzed anonymously)<br><br>**Animal Research (involving vertebrate animals, embryos or tissues)**<br>• Provide the name of the Institutional Animal Care and Use Committee (IACUC) or other relevant ethics board that reviewed the study protocol, and indicate whether they approved this research or granted a formal waiver of ethical approval<br>• Include an approval number if one was obtained<br>• If the study involved *non-human primates*, add *additional details* about animal welfare and steps taken to ameliorate suffering<br>• If anesthesia, euthanasia, or any kind of animal sacrifice is part of the study, include briefly which substances and/or methods were applied<br><br>**Field Research**<br><br>Include the following details if this study involves the collection of plant, animal, or other materials from a natural setting:<br>• Field permit number<br>• Name of the institution or relevant body that granted permission	
**Data Availability**<br><br>Authors are required to make all data underlying the findings described fully available, without restriction, and from the time of publication. PLOS allows rare exceptions to address legal and ethical concerns. See the PLOS Data Policy and FAQ for detailed information.	No - some restrictions will apply

Powered by Editorial Manager® and ProduXion Manager® from Aries Systems Corporation

A Data Availability Statement describing where the data can be found is required at submission. Your answers to this question constitute the Data Availability Statement and **will be published in the article**, if accepted.<br><br>**Important:** Stating 'data available on request from the author' is not sufficient. If your data are only available upon request, select 'No' for the first question and explain your exceptional situation in the text box.<br><br>Do the authors confirm that all data underlying the findings described in their manuscript are fully available without restriction?	
**Describe where the data may be found in full sentences. If you are copying our sample text, replace any instances of XXX with the appropriate details.**<br><br>• If the data are **held or will be held in a public repository**, include URLs, accession numbers or DOIs. If this information will only be available after acceptance, indicate this by ticking the box below. For example: *All XXX files are available from the XXX database (accession number(s) XXX, XXX.).*<br>• If the data are all contained **within the manuscript and/or Supporting Information files**, enter the following: *All relevant data are within the manuscript and its Supporting Information files.*<br>• If neither of these applies but you are able to provide **details of access elsewhere**, with or without limitations, please do so. For example:<br><br>*Data cannot be shared publicly because of [XXX]. Data are available from the XXX Institutional Data Access / Ethics Committee (contact via XXX) for researchers who meet the criteria for access to confidential data.*<br><br>*The data underlying the results presented in the study are available from (include the name of the third party*	ccording to Swedish legislation (https://etikprovningsmyndigheten.se/for-forskare/vadsager-lagen/) data cannot be made available for use beyond what has been approved<br>by the ethical review board. Therefore, the data cannot be made publicly available

Powered by Editorial Manager® and ProduXion Manager® from Aries Systems Corporation

and contact information or URL).<br>• This text is appropriate if the data are owned by a third party and authors do not have permission to share the data.<br><br>* typeset	
Additional data availability information:	

Powered by Editorial Manager® and ProduXion Manager® from Aries Systems Corporation

Manuscript Click here to access/download;Manuscript;Manuskript.docx

Novel hormonal therapy versus Standard of Care -- a registry-based comparative effectiveness evaluation for mCRPC-patients

Per Johansson, Professor
Department of Statistics, Uppsala University and Tsinghua University
Paulina Jonéus, PhD-student
Department of Statistics, Uppsala University
Sophie Langenskiöld, PhD
Department of Medical Sciences, Uppsala University

Corresponding author: Sophie.langenskiold@medsci.uu.se

Background

This paper presents results from one of the few comparative effectiveness evaluations of novel antiandrogen medications (NHT) against standard of care (SoC) for patients suffering from metastatic castrate-resistant prostate cancer (mCRPC).

Methods

The design and the analysis are published in a protocol before accessing outcome data. Two groups of patients are balanced on hundreds of important covariates measured before the prostate cancer diagnosis and up to the date of the prescription. While the design yields balance on the observed covariates, one cannot discard the possibility that unobserved confounders are not balanced. The unconfoundedness assumption is assessed by estimating placebo regressions on two health measures, not included in the design but added together with the outcome data after protocol publication.

Results

We find a substantial increase in mortality for patients prescribed with NHT rather than SoC. However, based on the results from one of the two placebo regressions, we cannot rule out that the difference in mortality may be due to confounding. However, using a bounding strategy of the effect, we can rule out that NHT reduces mortality compared to SoC. We can also rule out a positive effect on skeleton-related events (SRE) for the NHT patients.

Conclusions

Generally, the SoC for this group of patients is docetaxel. Given the substantially higher costs of NHT, the finding of no positive effects from NHT on both mortality and SRE is important.

More comparative studies, including studies analysing quality of life outcomes, are thus needed.

Introduction

Prostate cancer (PC) is the fifth leading cause of death in men worldwide, and almost all mortalities arise when patients have progressed to the advanced stage: metastatic castrate-resistant prostate cancer (mCRPC). Various treatment alternatives are available for these patients and in the last two decades, chemotherapy and new hormonal treatment (NHT) medications have revolutionized treatment (Basch et al., 2013; Beer et al., 2014; Loriot et al., 2015; Ryan et al., 2015; Tannock et al., 2004; Ye et al., 2017). However, to our knowledge, there is only one study comparing chemotherapy and NHT (Chowdhury et al., 2020). Unfortunately, in this study, the patients on docetaxel are more progressed at baseline than those on NHT. The authors do not describe how they adjust for these differences in their analyses.

This paper presents the results from a comparative effectiveness evaluation of two of these NHTs, abiraterone acetate (AA) in combination with prednisone and enzalutamide (ENZ), against standard of care (SoC). In our analysis, we estimate the average treatment effect on overall mortality. In addition, we estimate the effect on the prevalence of two secondary outcomes, skeleton-related events (SRE) and pain.

Data are collected from population registers administrated by the National Board of Health and Welfare (NBHW) and Statistics Sweden (SCB). The population is restricted to all men in the NBHW register with a PC diagnosis before 2017. The maintained assumption of the study design is that given observed pre-treatment covariates, the treatments (NHT or SoC) are

unconfounded. That is, we assume we observe the relevant covariates associated with the respective outcomes and, at the same time, determine the prescription. Mortality data from NBHW was added after the publication of the protocol; the protocol, including specified details concerning the design and analyses, is found in Jonéus et al. (2021).

Real-world data is increasingly accepted as evidence in the regulatory process (see, e.g., Alphs et al. (2015, 2014); Baumfeld et al. (2020); Egger et al. (2016); European Medicines Agency (2020)). A relevant question is therefore whether or not a protocol similar to the requirement for a randomized control trial can be pre-published. As yet, there is no consensus on the content requirement in such a protocol (Burns et al., 2022). As a potential input to the discussion, this paper illustrates that an observational study based on a pre-published protocol can encapsulate the same level of detail as a protocol for a randomized experiment.

The analysis concerns NHT usage in clinical practice from June 2015 to June 2018, which corresponds to the period when these drugs were reimbursed for mCRPC patients who had failed androgen deprivation therapy (ADT) and were not yet suited for docetaxel (pre-chemotherapy), or for patients who had failed docetaxel (post-chemotherapy). In June 2018, AA was additionally reimbursed as an add-on for high-risk metastatic hormone-sensitive prostate cancer (mHSPC) patients unsuited for docetaxel. Chemotherapy with docetaxel is found in previous, yet unpublished, qualitative work to be the SoC for this group of patients. Since docetaxel was approved in 2004 and there was no other first-line treatment for mCRPC until the introduction of the NHTs, this creates a unique situation for estimating the effect of the new NHT against SoC using historical data.

While the design yields balance on the observed covariates, one cannot discard the possibility that unobserved confounders are not balanced. Therefore, data from the national PC register (NPCR) was added after protocol publication, allowing us to objectively assess the unconfoundedness assumption using placebo regressions on two covariates measured before the NHT prescription. The PSA levels and the Gleason score are the two covariates judged by specialists as potential confounders.

We find a substantial increase in mortality for patients prescribed NHT rather than SoC. No differences in the morbidity outcomes were found. However, based on the results from the placebo regression on the Gleason score, we cannot rule out that the differences in mortality are due to confounding. However, an extended analysis allows ruling out that the estimates on mortality would have been negative and statistically significant in the presence of confounding, i.e., the opposite effects. Thus, this analysis allows us to rule out a reduction in mortality for patients prescribed NHT instead of SoC.

The remainder of the paper discusses the data and covariates in section 2. Section 3 provides a discussion of the design in Jonéus et al. (2021), together with a description of the analysis sample and the outcomes. Section 4 sets up the analysis model, and section 5 presents the results. The sensitivity analyses are conducted in section 6. Finally, the paper concludes in section 7. The study was approved by the Regional Ethical Review Board in Uppsala, Sweden (Dnr 2017/482).

Methods

Rich data concerning all patients' prostate cancer and disease progression is collected from the NBHW registers. The inpatient care register contains information about patients'

admission and discharge dates and information on the principal and secondary diagnoses for hospitalizations (using the ICD 10 classifications). The cancer register contains data on prostate cancer diagnosis. The pharmaceutical register contains the date of prescription and dispensing of drugs and their ATC class. Based on this information, we restrict the population to all men in Sweden with a PC diagnosis from 1986 to 2016.

The SCB data includes information on age, marital status, educational level, and country of birth, as well as a large set of covariates measuring security benefits, such as pensions, income, and sick leave. We collect data on socio-economic status at the year of diagnosis and the two preceding years. In a few cases of missing values, the average of the information from previous years is used for each individual. In addition, information on educational level is sometimes missing; in these cases, a five-nearest-neighbour approach is used to impute missing values.

Sample restrictions

The fact that the two NHTs were not offered by Swedish healthcare before being subsidized provides a unique opportunity for evaluating NHT against SoC in clinical practice. However, the comparison population should be as similar as possible to those prescribed an NHT, and the two populations should have been provided the same quality of healthcare. Finally, the outcome of the comparisons should not be affected by later being prescribed an NHT. To address the first two restrictions, we sample the SoC populations close in time to the NHT population. To address the latter restriction, we ensure that the SoC patients' follow-up period ends before NHTs are reimbursed, and that the NHT patients are unlikely to have been given SoC, which will be the case if NHT is prescribed not so distant in time from the date when given the diagnosis. Further details are provided in Jonéus et al. (2021).

Due to the sampling restrictions on the comparison population mentioned above, the population is restricted to a sample of 1,285 NHT-treated patients who were prescribed an NHT within 36 months of PC diagnosis. As we obtain mortality data until June 2020, we have a minimum follow-up period of two years and a maximum of five years for this group of patients. Therefore, to obtain a sufficiently large group of comparison patients, we sample all men with PC diagnosis from June 2008, to June 2010, giving us a sample of 19,456 patients. Thereby, also the patients can be observed for a maximum of five years without the risk that they are also subsequently treated with NHT, i.e., until 2015.

Only a few patients were prescribed an NHT within six months of diagnosis; most were prescribed it after more than 12 months. As no patients received treatment less than four months after diagnosis, the data can be described as a stratified sample with 33 strata, $w = 4, \ldots, 36$ months (see Figure A1 in Appendix A). Therefore, the analysis design considers each stratum separately.

Descriptive statistics on a sub-set of pre-diagnosis covariates for the two groups are displayed in Table 1. From the table, we can see that those prescribed NHT are older and have worse health than the comparison group, especially at the time of diagnosis but also to some extent 12 months before. The Elixhouser comorbidity index is based on ICD diagnostic codes and includes 31 comorbidities, summarized in a single numeric score (van Walraven et. al. 2009). We follow Menendez et al. (2014) and categorize each comorbidity score to one of four index groups where an index of 0 implies no comorbidities and an index of ≥5 implies several comorbidities and thereby the worst health condition. As seen in Table 1, the Elixhouser index indicates a larger difference in comorbidity status at the time of the diagnosis, where

NHT patients seem to have a worse health status, than when measured 12 months before diagnosis. The number of hospital visits (# hospital visits) shows a substantial statistical significant difference in the periods just before diagnosis, but not difference between six and 60 months before diagnosis. In short, there is a clear indication of more severe health progression in the NHT treatment group than in the comparison group before PC diagnosis.

Table 1: Means (standard deviations) of pre-diagnosis covariates of those prescribed NHT and comparisons (SoC), and the mean differences

Description	SoC	NHT	Diff.
Age at diagnosis	69.40 (9.16)	70.85 (8.45)	-1.45***
# Hospital visits 1 month bf. diagnosis	0.47 (0.84)	0.71 (1.10)	-0.23***
# Hospital visits 1-6 months bf. diagnosis	1.38 (2.36)	1.68 (3.15)	-0.30***
# Hospital visits 6-12 months bf. diagnosis	0.83 (1.96)	0.82 (2.60)	0.01
# Hospital visits 1-60 months bf. diagnosis	7.12 (11.00)	7.85 (23.72)	-0.73
Elixhouser index = 0, at diagnosis	0.53 (0.50)	0.44 (0.50)	0.09***
Elixhouser index = 1-4, at diagnosis	0.43 (0.50)	0.51 (0.50)	-0.08***
Elixhouser index ≥5, at diagnosis	0.03 (0.18)	0.05 (0.22)	-0.02***
Elixhouser index = 0, 12 months bf. diagnosis	0.62 (0.49)	0.54 (0.50)	0.08***
Elixhouser index = 1-4, 12 months bf. diagnosis	0.36 (0.48)	0.43 (0.50)	-0.07***
Elixhouser index ≥5, 12 months bf. diagnosis	0.02 (0.13)	0.03 (0.17)	-0.01**
Less than secondary school education	0.38 (0.49)	0.36 (0.48)	0.02
Secondary school education	0.39 (0.49)	0.39 (0.49)	-0.01
Living with a partner	0.65 (0.48)	0.64 (0.48)	0.01
Born in the Nordic countries	0.95 (0.22)	0.94 (0.23)	0.01

Number of individuals	19,456	1,285

*Standard deviations within parentheses. *p <0.1; **p <0.05; ***p <0.01.*

Variables measuring sickness progression after diagnosis

Six variables are used to measure sickness progression each month after the diagnosis until the month of treatment for those prescribed an NHT and for 36 months for the SoC patients (Jonéus et al., 2021). The latter is because we do not have access to the date regarding when SoC patients were administered SoC. However, information on these variables is only used up to the potential time when patients could serve as controls to patients on NHT, i.e., maximum of 36 months after PC diagnosis or until time of death (for more details, see Jonéus et al., 2021). The variables are (i) cumulative number of hospital visits, (ii) presence of metastases (ICD C77-C79), (iii) number of months with visceral metastases (ICD C78), (iv) number of months with skeleton metastases (ICD C795), (v) total number of collected daily doses of any antiandrogen, GnRH-agonist or GnRH-analog3, and (vi) the indicator of being prescribed only Bicalutamide or Bicalutamide+GnRH up until a specific month.

Design and outcome data

Jonéus et al. (2021) made use of the entropy balancing scheme suggested by Hainmueller (2012) for balancing the covariates between the two groups. Starting with uniform base weights, the reweighting scheme assigns a new scalar weight to each treated unit, ensuring that the reweighted comparison groups have the same mean (and higher moments) of the included covariates as the treated group. Then, the weights are recalibrated until the pre-defined balance across the groups is achieved. In order to adjust for the sickness progression from the time of diagnosis to prescription, the balancing is performed separately for each of

the 33 strata. Thus, for each stratum, we adjust for covariates measured at the time of diagnosis and for the stratum-specific covariates capturing the sickness progression.

The complete set of covariates includes interactions and second-order terms, in addition to the linear terms. In addition to balancing the means, we also balance the variances of the main covariates between the two groups. This results in a balance of up to 56 covariates, depending on the stratum.

Outcome data

We have one primary and two secondary morbidity outcomes. The primary outcome is all-cause mortality (DEAD); the two secondary outcomes are SRE and PAIN. SRE is an indicator of a skeleton-related event, and PAIN is an indicator of severe pain. The evaluation period is 24 months after treatment.

DEAD is an indicator variable of zero if the patient is alive at the end of each 30 days and one if the patient died during that specific period. Patients are assumed to suffer a skeleton-related event (SRE) if they experience a hospitalization because of pathologic fracture (ATC codes M485, M495, M844, and M907) or spinal cord compression (G550, G834, G952, G958, G959, and G992) (Parry et al., 2019). The SRE indicator is one for every 30 days of such hospitalizations, and zero for other periods.

Patients are assumed to suffer severe pain if they receive prescriptions for neuropathic pain, i.e., opiates in combination with tramadol and paracetamol (ATC-codes N02AA, N02AX02, and N02BE01). The PAIN indicator is one for each 30-day period in which the patient has received such a prescription, and zero for the other periods.

Mortality data were added after the publication of the pre-analysis plan (Jonéus et al., 2021). In this data, 9,389 of the 20,757 patients (45%) in the sample have a date of death. Of these 9,389 patients, 3,832 have the PC diagnosis registered as the main cause of death (i.e., ICD C61).

Analysis and estimation

Let $Y_{i,m}$ denote any of the three outcomes observed for patient *i* in month m. Let $\mathrm{T}_i = 1$ if the patient is prescribed NHT and $\mathrm{T}_i = 0$ otherwise. We regress $Y_{i,m}$ on T_i and stratum indicators (see Appendix C for details). The monthly effects are estimated using the entropy balancing weights, and standard errors are estimated using a robust covariance estimator (see, e.g., Huber, 1967). The estimated confidence intervals are adjusted for multiple testing using Bonferroni correction, and we let the overall significance level be 5%. With one primary and two secondary outcomes, the significance level of the single outcome is 1.67% ($= 100 * 0.05/3$).

Note that every comparison individual has a weight (could be zero, though) in each of the 33 strata, that is, the month after diagnosis when the NHT patients were prescribed the drug. If we had data on mortality when designing the study, SoC patients who died any time before month 36 would have been removed from consideration. Around 8% of the comparisons in each stratum have died before, or in, this monthly stratum. The entropy weights were recalculated after censoring patients in the comparison group at the time of death. As the results using these new entropy weights are all qualitatively similar (see Appendix A) to the results based on the pre-specified design, we present the result with the original design below for transparency.

Results

The mortality analysis results are presented in sub-section 5.1, and the morbidity outcomes are given in sub-section 5.2. Finally, the last sub-section presents results from the separate analysis of cases with prescriptions of NHT given early or late after diagnosis.

Mortality

The results on the incidence of mortality for the 24 periods of 30 days are presented in Figure 1, while effects from a survival analysis are presented in Figure 2. In the survival analysis, we estimate a discrete time Cox regression model.[1] In this analysis, we have a follow-up period of 69 months for NHT patients and 82 for SoC patients.

Figure 1 displays the point estimates, the 95% Bonferroni corrected confidence intervals, and overall mean mortality on 24 periods of 30 days, in the following denoted months. A clear pattern of higher mortality is seen in patients if prescribed NHT against SoC. Further, the 95% confidence intervals do not cover zero 16 times out of 24. The average increase in mortality over the 24 months is 2.3 percentage points, or 64% when normalizing with the overall mean mortality for the two groups (0.0357).

<Figure 1 here>

Figure 1: The effect on mortality of NHT against SoC. Estimates, 95% Bonferroni confidence intervals, and overall mortality in each month (x)

[1] For the discrete-time Cox regression model and the results from the analysis, see Table A1 in Appendix A.

The estimated survival functions for the two groups is displayed in Figure 2a, while the corresponding hazard functions, with confidence intervals, are given in Figure 2b. The figure show that the monthly hazard is around 5 percent for the NHT patients and around 3.5% for SoC. These results confirm those from the incidence analysis: a substantial difference in mortality in months 8 to 24. However, from this graph, we can also see that this difference persists for 60 months.

<Figure 2a> <Figure 2b>

Figure 2: The estimated survival function and conditional probability of dying each month (i.e., the monthly hazard) for NHT (—) and SoC (- - -) patients up to 82 months after a potential drug prescription of NHT

Skeleton-related events and severe pain

We observed 760 unique patients (4%) suffering from an SRE, 12% in the NHT group, and 3% in the SoC group. Only 376 (2%) unique patients have severe pain, according to our definition of PAIN, and in the group of NHT patients, the proportion is 0.2%.

However, we only observed an indication of an SRE or PAIN when the patient was alive. As the mortality is higher for the NHT group, we are consequently more likely to observe any of these morbidity outcomes for SoC patients than for NHT patients. To manage this problem, we estimate the bounds of potential effects. First, we let all patients who die have either a no morbidity or a morbidity outcome. For the SRE, this means letting SRE=0 or SRE=1. Then, since the mortality with NHT is observed to be higher than with SoC, the first case with SRE=

0 provides a lower bound estimate of the effectiveness of NHT versus SoC in preventing SRE, while the second with SRE=1 one provides an upper bound.

The results from these analyses on SRE are presented in Figure 3. We find no differences in effect on SRE if we treat all who died as not suffering from an SRE. On the other hand, if all who died suffered from an SRE, then the NHT patients would have had less SRE if they had instead had SoC. This result allows us to rule out a positive effect on SRE for the NHT patients if they had been prescribed SoC instead. According to Wong et al. (2019), most mCRPC patients who die suffer from bone metastases. As SRE is a complication of bone metastases, it is possible that the 'true' differences are closer to the upper bound results than the lower.

As only 2% of the patients have severe pain and the proportion is only 0.2% among NHT patients, the effects are very imprecisely estimated. Nevertheless, for transparency, the results from these analyses are presented in Figure A3 and A4, Appendix A.

<Figure 3a> <Figure 3b>

Figure 3: Effects on SRE of NHT against SoC. Estimates and 95% Bonferroni corrected confidence intervals. Lower (left panel) and upper (right panel) bounds of potential effect

Effects on early and late prescription

We do the analysis presented in Section 5.2 separately for those with less than the median time (less than 20 months) to NHT prescription from PC diagnosis, and longer or equal to the median time (more than or equal to 20 months) on the three outcomes. The results indicate that the effect could be substantially greater for those with early rather than late prescriptions.

However, as almost all confidence intervals overlap zero, no firm conclusion can be made (see Figure A5 in Appendix A). The results for the two morbidity outcomes did not show any clear pattern but are included for transparency (see Figures A6 and A7).

Sensitivity analyses

We also matched data from NPCR to the analysis sample when adding mortality data. These data contain more detailed information on patients' health with regard to the PC. Three covariates, judged by specialists to be important confounders and measured at the time of PC diagnosis, are used: the PSA levels (SPSA), Gleason score (GleasSa), and metastases (Mstad).

The validity of the design is assessed by estimating ʻplacebo' effects on these covariates. If there are statistically significant effects on these covariates, available data from the population register may not be sufficient to control for confounding bias.

One crux with this analysis is that the NPCR does not have full coverage, and there are partly missing data on the covariates. Therefore, before turning to the placebo regression, we analyse the missing data problem in the following sub-section.

Missing data

There are 1,083, 1,367 and 14,923 missing observations for SPSA, GleasSa and Mstad, respectively. The placebo regressions are valid if the missing observation can be treated as missing at random. From the analysis of missingness (see Appendix B), we conclude that Mstad is not a valid proxy outcome. Except for stratum 4, 5, and 26 for GleasSa, we can treat the observations as missing at random. As the degree of missingness for SPSA and remaining

strata for GleasSa are low, we remove patients with missing observations from the sensitivity analyses.

Placebo regression

We use the same analysis as in the main analysis but instead regress the two proxy outcomes, i.e., SPSA or GleasSa, on the NHT indicator. As the entropy weights are at the stratum level, we estimate the association at the stratum level (see Appendix C). This means we get 33 and 30 (as we exclude stratum 4, 5, and 26) estimates of the association for the SPSA and GleasSa, respectively. The proxy outcomes are standardized to have zero mean and unit variance per strata. The results from these analyses are shown in Figure 4.

<Figure 4a> <Figure 4b>

Figure 4: Results from the placebo regression on SPSA (left) and Gleason (right). Estimate and 95% Bonferroni corrected confidence interval for each month

The left panel shows the results for SPSA, and the right the results for GleasSa. There are no differences between the two groups with regard to SPSA. However, NHT patients are seen to have between 0.27 to 0.96 standard deviation higher levels on the GleasSa than SoC patients. Most of these estimates are also statistically significant. Figure A10 in Appendix A reports qualitatively similar results from the placebo regressions with the updated weights.

The Gleason score is a confounder in the analysis if it is also associated with the outcome, conditional on the covariates. To this end, we recalculate the weights, including GleasSa. The results from this analysis are presented in Figure A11 in Appendix, and are similar to the results from the main analysis. All estimates are positive, with one exception for the first

month, and the majority of these are statistically significant. The overall picture of increased mortality from NHT is thus not changed due to the imbalance in the GleasSa.

There could, of course, be other confounders pointing to why a firm conclusion of increased mortality from being prescribed NHT could be questioned. Given the differences in cost of the two types of treatment, it is, however, interesting to consider whether we can rule out a reverse effect on mortality for *each stratum*. The substantial imbalance of 0.96 standard deviations found for stratum six is used in this derivation. Appendix C details a framework for this analysis, but the idea and results from the analysis are provided in the following two paragraphs.

The idea is as follows: using the imbalance of 0.96 standard deviations, we derive the smallest possible association an unobserved confounder needs to have with mortality to obtain a statistically significant and reversed sign of the mortality estimate. We then compare this derived association with the estimated associations between (1) mortality and age, and (2) mortality and number of months with skeleton metastases (metastases) in the *unadjusted sample*. If the estimated association between mortality and these two variables is larger than the derived minimum, we cannot rule out is the existence of an 'unobservable' that could reverse the inference. If, on the other hand, the estimated association between mortality and these two variables is smaller than the derived minimum, we believe it is safe to rule out a reverse effect. The reason for this is that both age and metastases are highly associated with mortality, and, at the same time, the two covariates are imbalanced in the *unmatched sample* (see Table A2 in Appendix A). It is hard to imagine an 'unobservable' being more imbalanced and highly associated with mortality than these two covariates.

The result from this analysis is shown in Table 2. The derived lower limit of a potential reverse effect is given in column (2). An association between an unobserved confounder and mortality, larger than the derived lower limit, could yield a statistically significant negative effect on mortality from NHT compared to SoC; in other words, a statistically significant reduction in mortality on NHT compared to SoC. The estimated association (standard errors in parentheses) of mortality with age and metastases are presented in columns 3 and 4, respectively. The only larger estimate is in the first month for metastases (see column 4). Thus, the inference could potentially be changed but only for this month. The lower limit is substantially larger than the estimates for all other comparisons.

At a very late stage, we were informed that the grading making up the Gleason score was changed in November 2014 (see, e.g., Epstein, 2014, 2016). This means that the Gleason score of the SoC patients is graded according to the old system, whereas some of the NHT patients are graded according to the updated grading system. We, therefore, need to evaluate whether or not the modification is the main reason for the imbalance seen in Figure 4b.

The result from this analysis indicates that the imbalance is not only a consequence of the modification. For details on this evaluation, see Appendix A.

Table 2: The derived lower limit of a potentially reversed effect for each month and the estimated association (standard errors in parentheses) between mortality and metastases and age, respectively

Month	Lower limit ($\hat{\delta}_m^l$)	Metastases	Age
1	0.0054	0.00079 (0.0013)	0.00031 (0.00042)
2	0.0210	0.00205 (0.0013)	0.00034 (0.00037)

3	0.0273	0.00166 (0.0012)	0.00144 (0.00040)
4	0.0292	0.00225 (0.0013)	0.00188 (0.00040)
5	0.0369	0.00212 (0.0013)	0.00123 (0.00039)
6	0.0304	0.00279 (0.0013)	0.00183 (0.00037)
7	0.0265	0.00225 (0.0013)	0.00163 (0.00037)
8	0.0427	0.00362 (0.0013)	0.00201 (0.00039)
9	0.0488	0.00336 (0.0013)	0.00229 (0.00038)
10	0.0511	0.00284 (0.0014)	0.00202 (0.00039)
11	0.0381	0.00339 (0.0013)	0.00205 (0.00039)
12	0.0428	0.00226 (0.0012)	0.00156 (0.00036)
13	0.0528	0.00334 (0.0014)	0.00149 (0.00036)
14	0.0500	0.00452 (0.0014)	0.00248 (0.00041)
15	0.0722	0.00380 (0.0014)	0.00204 (0.00039)
16	0.0423	0.00390 (0.0013)	0.00176 (0.00038)
17	0.0650	0.00419 (0.0014)	0.00158 (0.00038)
18	0.0641	0.00461 (0.0014)	0.00160 (0.00037)
19	0.0652	0.00409 (0.0014)	0.00181 (0.00039)
20	0.0581	0.00343 (0.0014)	0.00230 (0.00040)
21	0.0346	0.00326 (0.0014)	0.00202 (0.00039)
22	0.0714	0.00232 (0.0014)	0.00215 (0.00040)
23	0.0430	0.00279 (0.0014)	0.00184 (0.00038)
24	0.0634	0.00304 (0.0014)	0.00253 (0.00041)

Discussion

The main result from the paper is that we can rule out a reduction in mortality and skeleton-related events for patients if prescribed NHT rather than being given standard-of-care treatment (SoC). The SoC for this group of patients is, in general, chemotherapy with docetaxel (cf. Johansson et al., 2021). The results on mortality are, to some extent, in agreement with the results in Chowdhury et al. (2020), who found no statistically significant difference in mortality three years after treatment for patients on NHT against those on docetaxel. However, in Chowdhury et al. (2020), patients on docetaxel are less healthy at baseline than those on NHT. It is unclear how they adjust for the differences in health at baseline in their analyses.

As we can rule out positive effects on both mortality and SRE, it is difficult to justify the high extra treatment cost of NHT. Rough estimates indicate that the cost for SoC would amount to 400 to 700 Euro for the 6-10 cycles of docetaxel, whereas the cost for NHT would vary between 25,000 and 33,000 Euro during this same period. On the other hand, as docetaxel is given by injection, the cost to the healthcare system is reduced with NHT. Therefore, to make a more relevant comparison of the costs of the two drugs, these costs should also be estimated.

The results are obtained when these drugs were made available for a population of men with prostate cancer. As a consequence of using historical controls, the population is restricted to patients prescribed NHT relatively shortly after diagnosis. Potentially, the effect may differ for other populations. There could, of course, be positive effects on outcomes not examined in this study. One such variable could be quality of life. Thus a full-blown cost-effectiveness evaluation needs results from more studies, including studies on outcomes not examined in the paper.

Acknowledgments

The authors thank seminar participants at Uppsala University for valuable comments and the Swedish Dental and Pharmaceutical Benefits Agency (TLV) for valuable fundings.

References

Alphs, L., Benson, C., Cheshire-Kinney, K., Lindenmayer, J. P., Mao, L., Rodriguez, S. and Starr, H. L. Real-world outcomes of paliperidone palmitate compared to daily oral antipsychotic therapy in schizophrenia: a randomized, open-label, review board-blinded 15-month study. The Journal of Clinical Psychiatry 2015;76:554-561.

Alphs, L., Mao, L., Rodriguez, S. C., Hulihan, J. and Starr, H. L. Design and rationale of the paliperidone palmitate research in demonstrating effectiveness (pride) study: a novel comparative trial of once-monthly paliperidone palmitate versus daily oral antipsychotic treatment for delaying time to treatment failure in persons with schizophrenia. The Journal of Clinical Psychiatry 2014;75:1388-1393.

Basch, E., Autio, K., Ryan, C. J., et al. Abiraterone acetate plus prednisone versus prednisone alone in chemotherapy-naive men with metastatic castration- resistant prostate cancer: patient-reported outcome results of a randomised phase 3 trial. The Lancet Oncology 2013;14(12):1193-1199.

Baumfeld, A., Caubel, P., Azoulay, L. and Dreyer, N. Trial designs using real-world data: The changing landscape of the regulatory approval process. Pharmacoepi- demiology and Drug Safety 2020;29:1201-1212.

Beer, T. M., Armstrong, A. J., Rathkopf, D. E., et al. Enzalutamide in metastatic prostate cancer before chemotherapy. New England Journal of Medicine 2014;371(5):424-433.

Burns, L., Roux, N. L., Kalesnik-Orszulak, R., et al. Real-world evidence for regulatory decision-making: Guidance from around the world. clinical therapeutics. Research Synthesis Methods 2022;44:420-437.

Chowdhury, S., Bjartell, A., Lumen, N., et al. Real-world outcomes in first-line treatment of metastatic castration-resistant prostate cancer: the prostate cancer registry. Targeted Oncology 2020;15(3):301-315.

Egger, M., Moons, K. G., Fletcher, C. and 4, G. W. GetReal: from efficacy in clinical trials to relative effectiveness in the real world. Research Synthesis Methods 2016;7:278-281.

Epstein, J. I., Egevad, L., Amin, M. B., Delahunt, B., Srigley, J. R. and Humphrey, P. A. The 2014 International Society of Urological Pathology (ISUP) consensus conference on Gleason grading of prostatic carcinoma. The American Journal of Surgical Pathology 2016;40(2): 244-252.

European Medicines Agency, Pharmaceutical Strategy for Europe, HMA-EMA Joint Big Data Taskforce-summary report, 2020. EMA/105321/2019. (https://www.ema.europa.eu/en/documents/minutes/hma/ema-joint-task-force-big-data-summary-report_en.pdf) Accessed 23 November 2022.

Hainmueller, J. Entropy balancing for causal effects: A multivariate reweighting method to produce balanced samples in observational studies. Political Analysis 2012;20(1): 25-46.

Huber, P. J. The behavior of maximum likelihood estimates under nonstandard conditions, in ‘Proceedings of the Fifth Berkeley Symposium on Mathematical Statistics and Probability’, Vol. I, University California Press, Berkeley, CA, 1967. pp. 221-233.

Johansson, P., Jonéus, P. and Langenskiöld, S. Study protocol for a comparative effectiveness evaluation of abiraterone acetate against enzalutamide: a longitudinal study based on Swedish administrative registers. BMJ Open 2021;11(10);e052610.

Jonéus, P., Johansson, P. and Langenskiöld, S. A study protocol for a comparative effectiveness evaluation of antiandrogenic medications against standard of care. arXiv preprint 2021 arXiv:2110.02698.

Loriot, Y., Miller, K., Sternberg, C. N., et al. Effect of enzalutamide on health-related quality of life, pain, and skeletal-related events in asymptomatic and minimally symptomatic, chemotherapy-naive patients with metastatic castration- resistant prostate cancer (prevail): results from a randomised, phase 3 trial. The Lancet Oncology 2015;16(5): 509-521.

Ryan, C. J., Smith, M. R., Fizazi, K., et al. Abiraterone acetate plus prednisone versus placebo plus prednisone in chemotherapy-naive men with metastatic castration-resistant prostate cancer (cou-aa-302): final overall survival analysis of a randomised, double-blind, placebo-controlled phase 3 study. The Lancet Oncology 2015;16(2):152-160.

Tannock, I. F., De Wit, R., Berry, W. R., et al. Docetaxel plus prednisone or mitoxantrone plus prednisone for advanced prostate cancer. New England Journal of Medicine 2004;351(15):1502-1512.

Wong, S. K., Mohamad, N. V., Giaze, T. R., Chin, K. Y., Mohamed, N. and Ima- Nirwana, S. Prostate cancer and bone metastases: The underlying mechanisms. International Journal of Molecular Sciences 2019;20:2587.

Ye, D., Huang, Y., Zhou, F., et al. A phase 3, double-blind, randomized placebo-controlled efficacy and safety study of abiraterone acetate in chemotherapy-naive patients with mCRPC in China, Malaysia, Thailand and Russia. Asian Journal of Urology 2017;4(2):75-85.

Click here to access/download;Figure;Figure 1.jpg

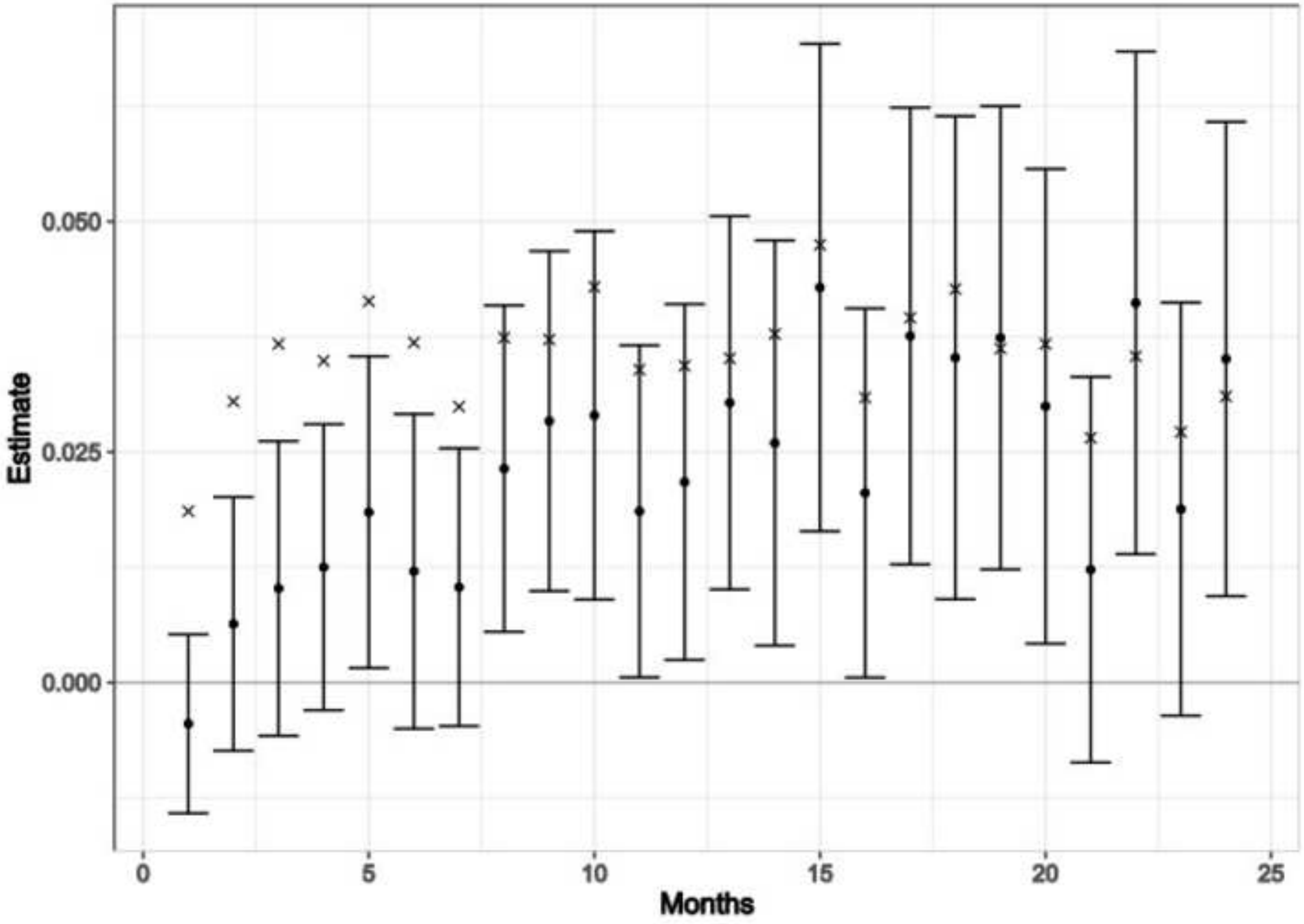

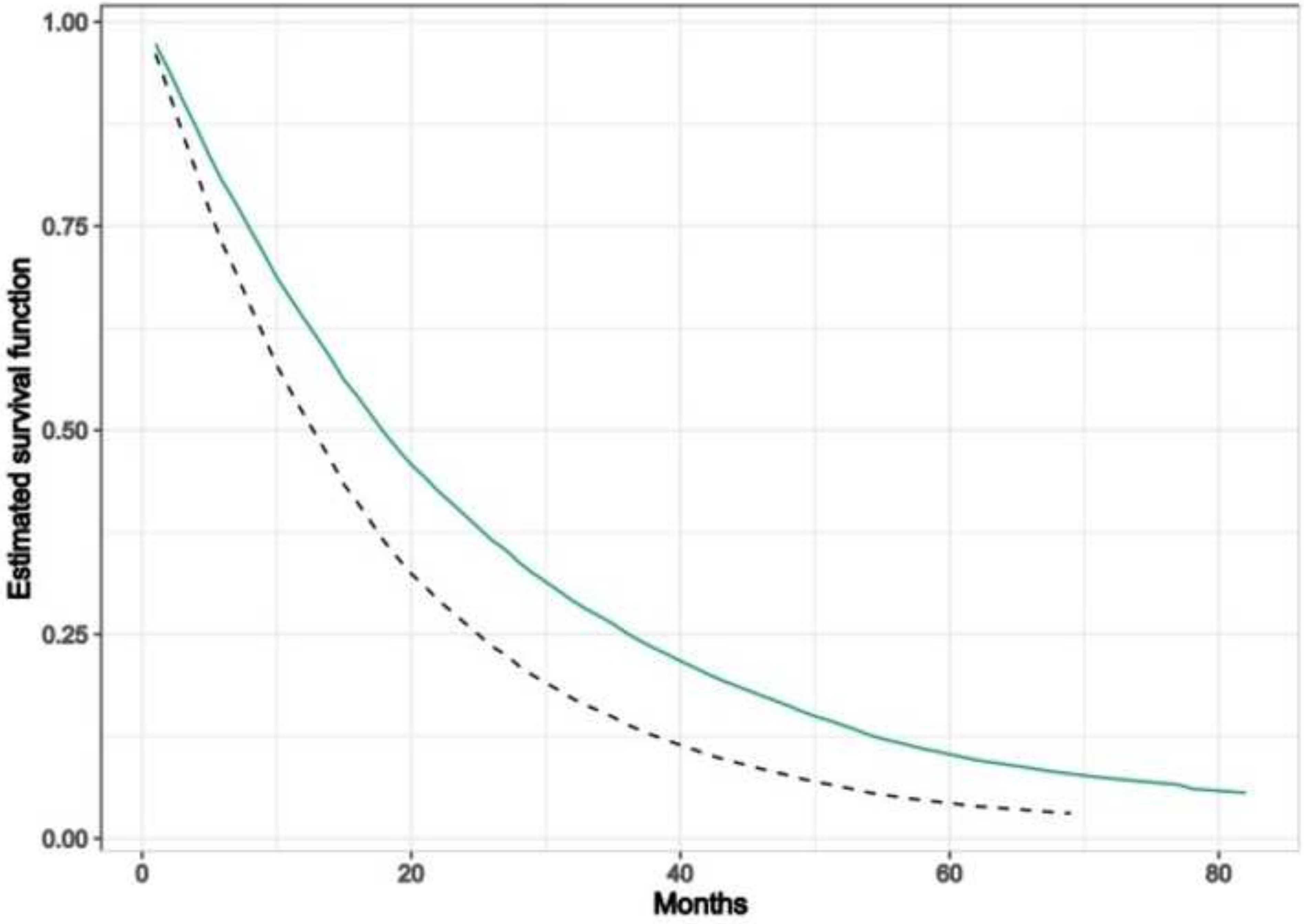
Estimated survival function
1.00
0.75
0.50
0.25
0.00
0
20
40
60
80
Months

 Click here to access/download;Figure;Figure 2b.jpg

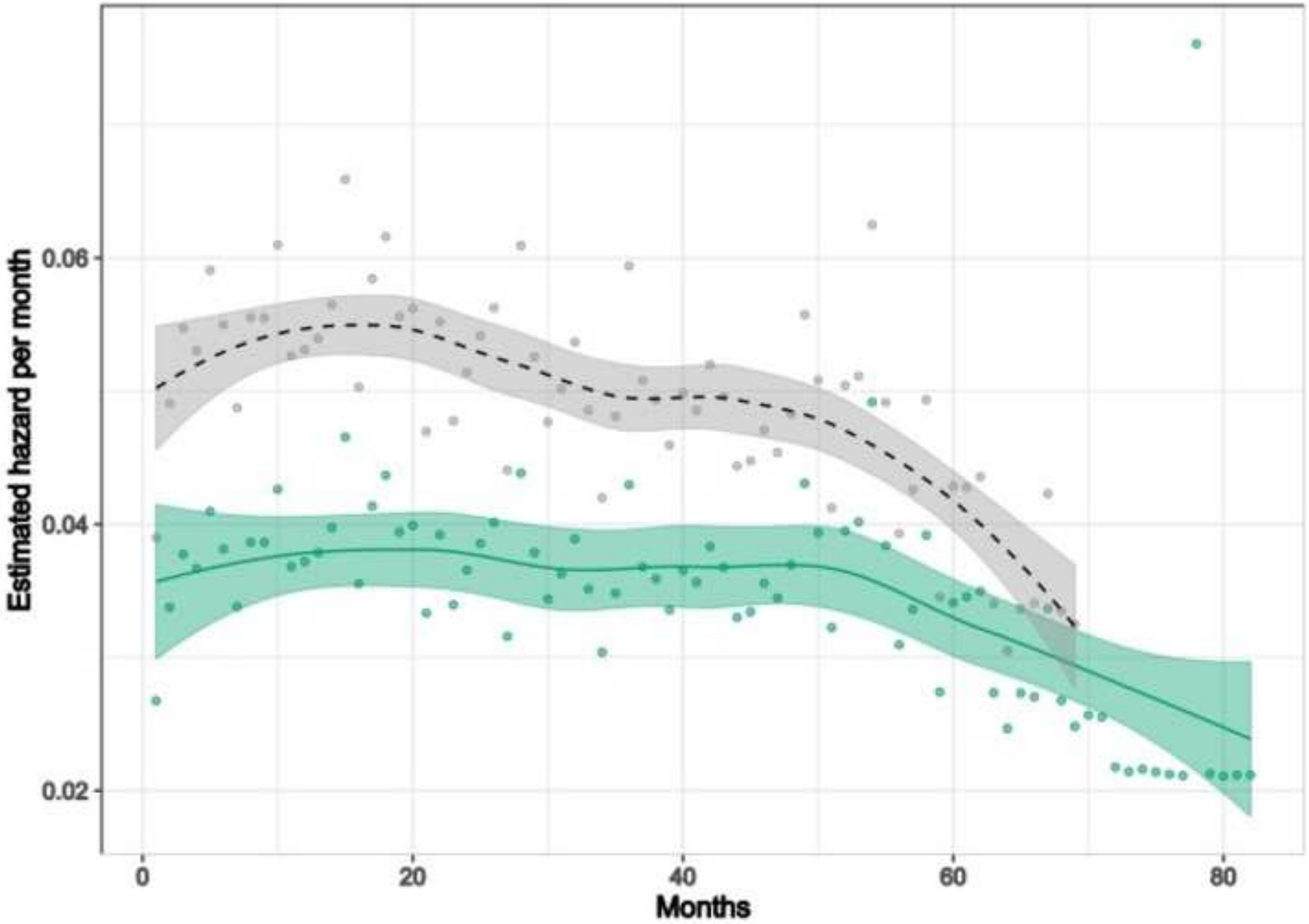

Click here to access/download;Figure;Figure 3a.jpg

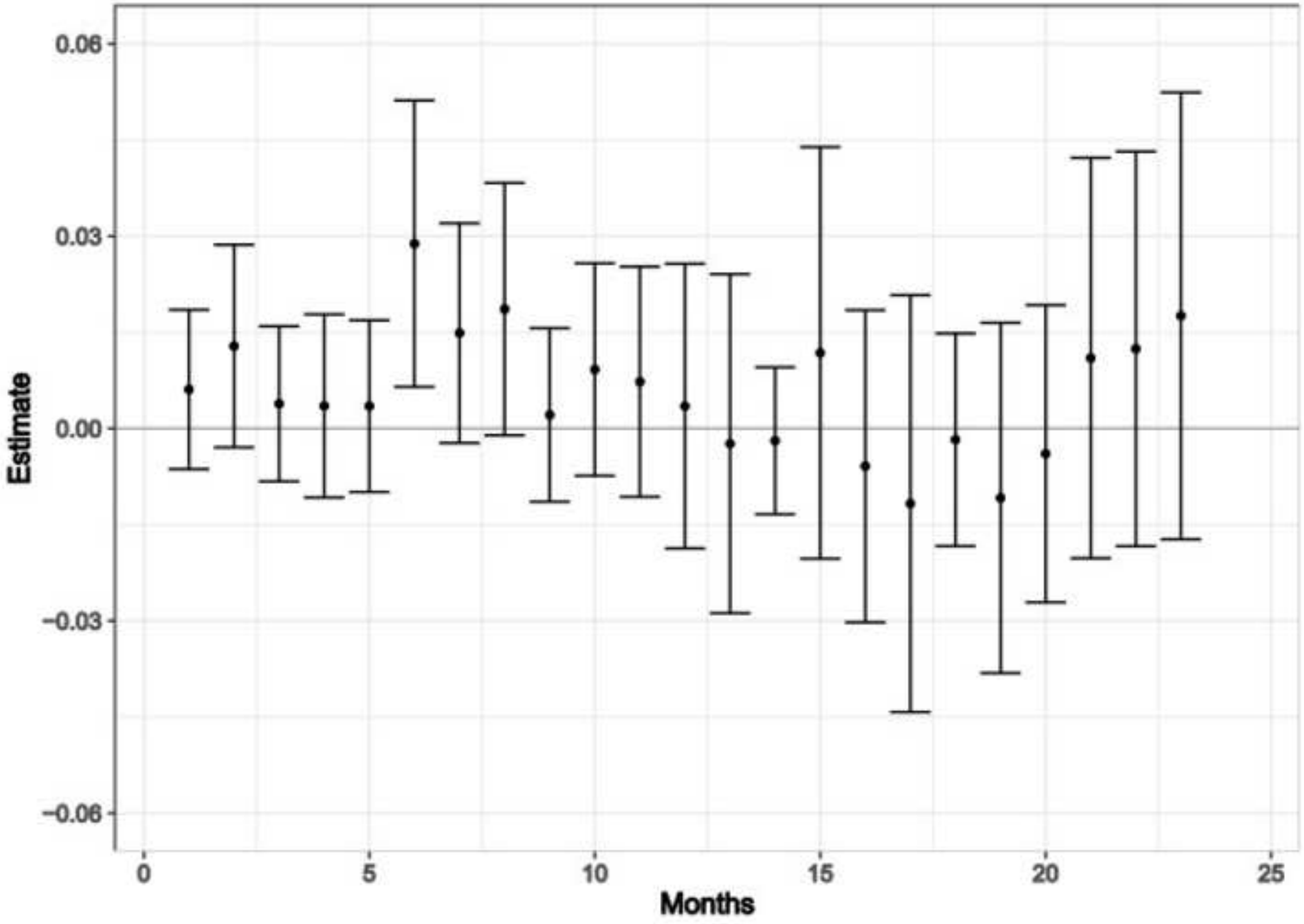

Click here to access/download;Figure;Figure 3b.jpg

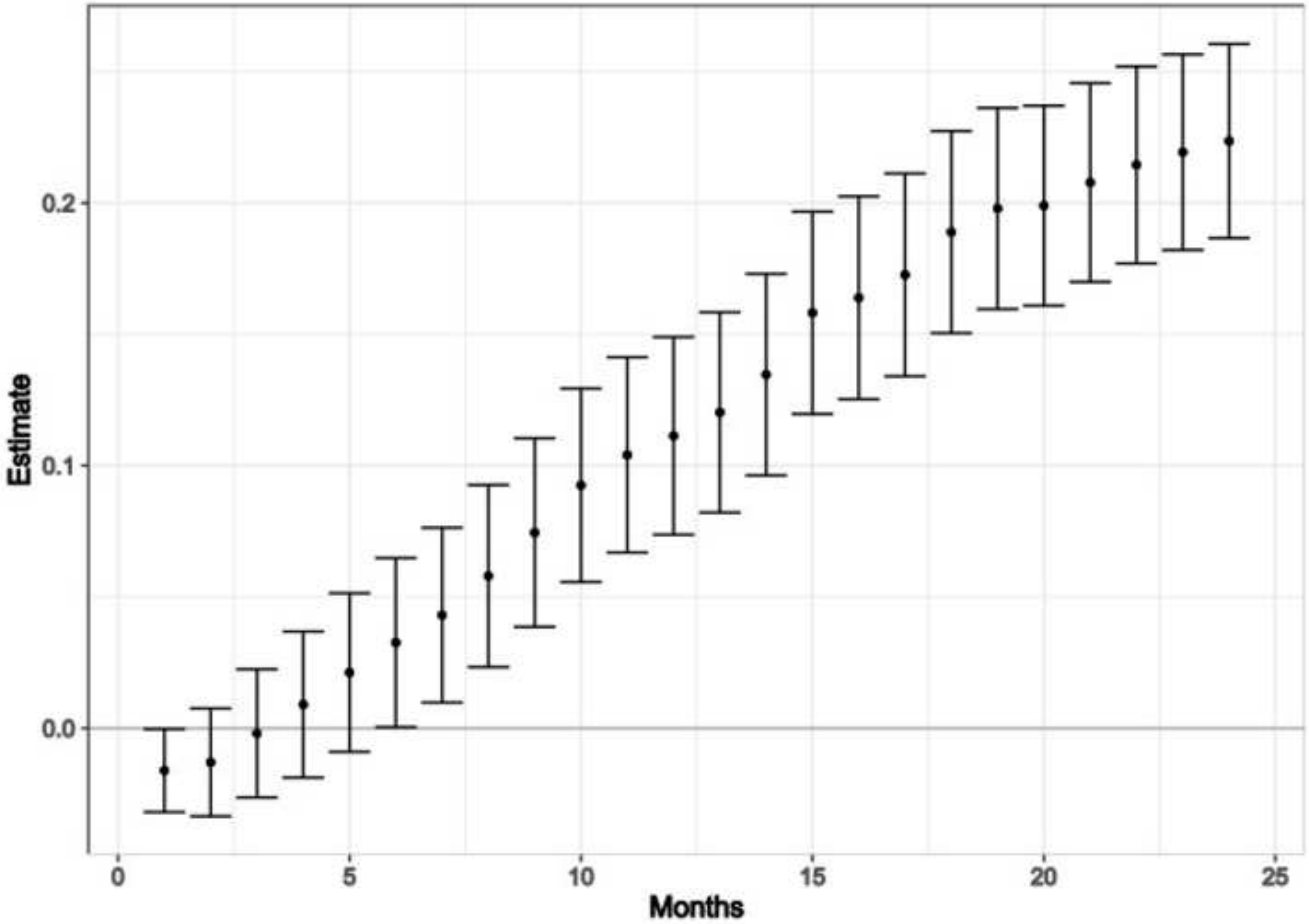

Click here to access/download;Figure;Figure 4a.jpg

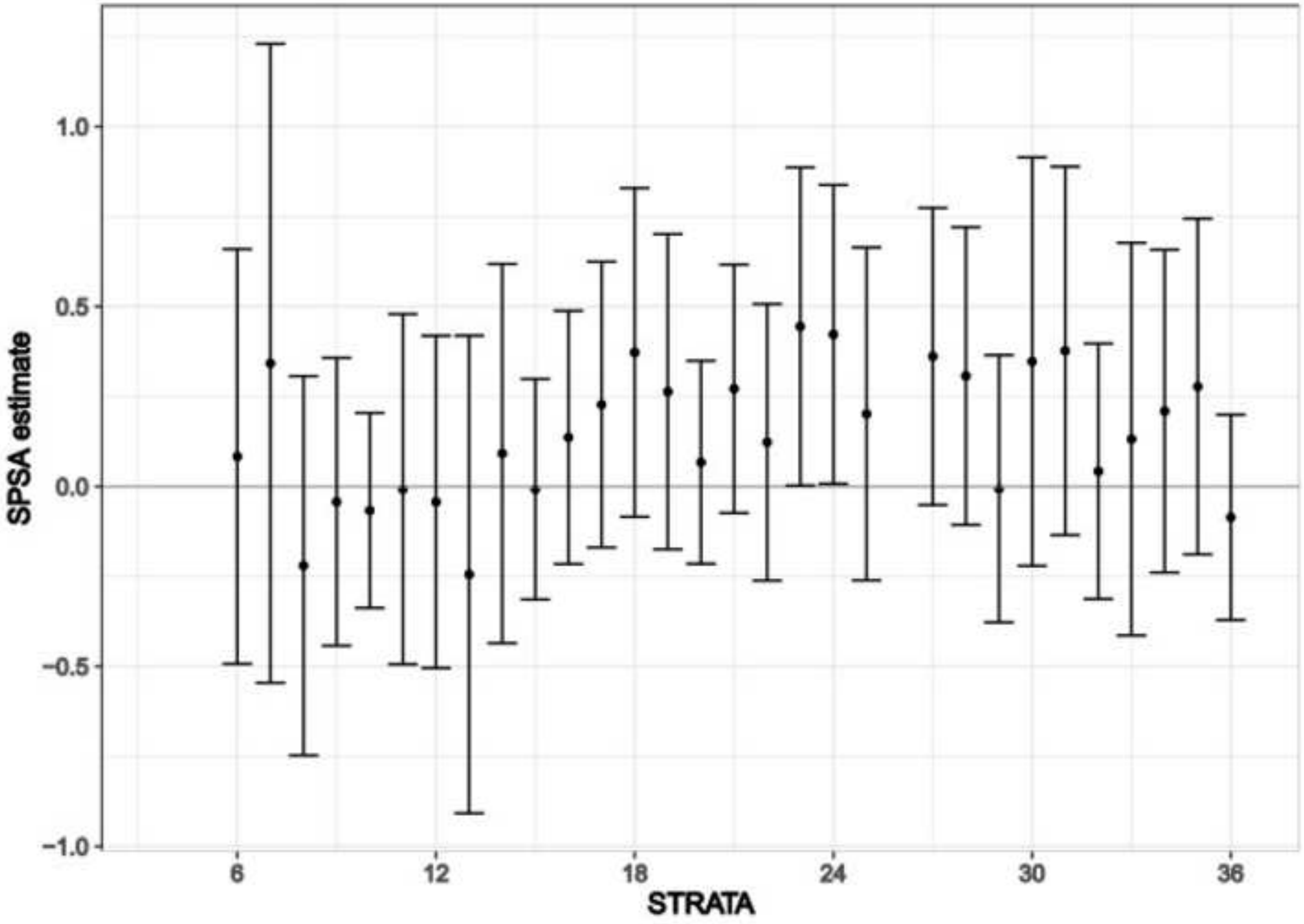

Cover Letter
Click here to access/download;Figure;Figure 4b.jpg

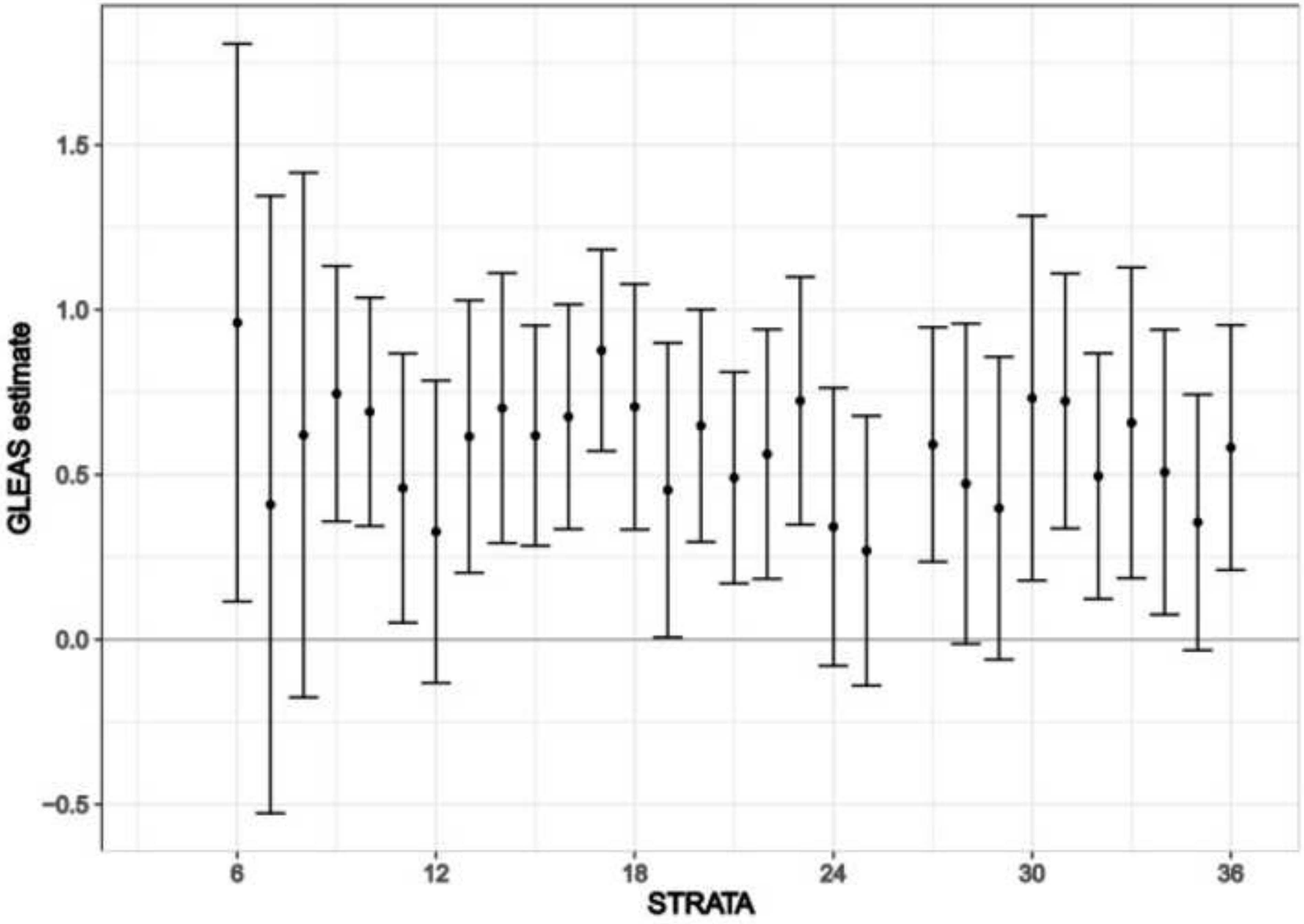

Click here to access/download
Supporting Information
Protocol.pdf
