# Supplementary material for: Novel hormonal therapy versus standard of care—A registry-based comparative effectiveness evaluation for mCRPC-patients
Source: PLoS One. 2024 Feb 14;19(2):e0290833. doi: 10.1371/journal.pone.0290833 (PMC10866493; doi:10.1371/journal.pone.0290833)
Supplement: Attachment — Submitted filename: Addressed Comments.doc [file pone.0290833.s019.doc]

|  | **Editor Comments:** | **Our comments:** |
| --- | --- | --- |
| 1 | Follow the body formatting guidelines. | We apologize for our failure to meets PLOS ONE’s style requirements, and have now made the below changes:   (1) We use Vancouver citing and referencing requirements (2) We have changed the format, citation and naming of the figures. (3) We have changed the citation, titles, and legends of tables. (4) We have changed the font size of the headings. (5) We have moved the acknowledgments to the end. (6) We have added supplementary information in the manuscript   However, as these changes are many, they have not all been highlighted in yellow in the attached manuscript. |

|  | **Reviewer Comments:** | **Our comments:** |
| --- | --- | --- |
|  | Abstract: The abstract provides a clear understanding of the study objectives and methodology. there are a few areas that could benefit from further clarification and improvement. Specifically: | |
| 1 | Line 14: The statement "one of the few comparative effectiveness evaluations" needs to be supported by evidence. It would be helpful to mention any existing studies that have evaluated the comparative effectiveness of NHT and SoC in mCRPC. | In the manuscript (see line 44-49, we have now summarized the two previous comparative effectiveness studies that we are aware of which study mortality differences. |
| 2 | Line 28: The abstract states that there is a substantial increase in mortality for patients prescribed with NHT, but it would be helpful to quantify this increase and provide a measure of effect size or a confidence interval. | We are evaluating the effects for many periods, and these effects cannot straightforwardly be translated to a single magnitude together with a correct confidence interval. We also prefer the non-parametric hazard model estimated with fewer assumptions, which do not provide the point estimated provided by a proportional hazard model. However, we now have added 64% as a point estimate which is the average increase in mortality over the 24 months evaluated at the overall mean mortality for the two groups (see lines 27 and 265). |
| 3 | Line 32: The statement "We can also rule out a positive effect on skeleton-related events (SRE) for the NHT patients" should be supported by specific findings or statistical results. | Line 32 is revised and reframed (see lines 31-33).  The statement is based on the finding in Wong et al. (2019) that most mCRPC patients who die suffer from bone metastases. It is clear that if all mCRPC patients suffer from bone metastases, we can rule out a positive effect from NHT from our result presented in our figure 3.  However the statement is too strong and we apologise. As a consequence we removed line 295 in the document were we incorrectly wrote “This result allows us to rule out a positive effect on SRE for the NHT patients if they had been prescribed SoC instead.’  In addition, we changed the wording in the concluding section (lines 435-436) and discussed the result in a new summary section suggested below (see lines 322-328). |
|  | Introduction: The introduction provides an overview of the study objectives and methodology. However, there are a few areas that could benefit from further clarification and improvement. Specifically: | |
|  | Line 47: The statement that "there is only one study comparing chemotherapy and NHT" should be supported by a citation to validate this claim. | At the time of initiating this research, a structured literature review was conducted Only one study was found evaluation chemotherapy against NHT in terms of survival difference. Since then, we became aware of a second study comparing the same treatments. Therefore, we conclude that the two interventions which are claimed to be the alternative 1^st^ line treatment for patients with mCRPC have only been rarely evaluated. We have clarified this in the revised manuscript (se lines 44-49).  However as there is always a possibility of missing relevant literature, and then especially grey literature we stated “to our knowledge”. We understand that this can be misinterpreted as not having conduced a structured review. In the revised document, it is clarified that a structured literature review was conducted (line 44). |
|  | Line 49: The authors mention differences in baseline characteristics between patients on docetaxel and NHT but do not clearly state whether these differences were adjusted for in their analyses. This should be clarified. | The statement on this study refers to Chowdhury et al., (2020) and not our study. As a consequence line 49-51 are revised (see lines 47-49). |
|  | Method: The methods section is well-structured and provides a comprehensive overview of the research design. However, there are a few areas that require clarification and improvement. Specifically: | |
| 1 | The authors should consider expanding the methods section to provide more context and justification for the chosen entropy balancing methodology. This would enhance the understanding of readers who may not be familiar with the statistical techniques. | The method is explained in detail in the pre-analysis Jonéus et al. (2021). For this reason, we did not provide any details on intuition for the method in this paper. We apologize. In this version we have stressed to provide an intuition by comparing it to propensity score matching (see lines 192-202). |
| 2 | The rationale behind the selection of a 36-month window for NHT-treated patients and the choice of June 2008 to June 2010 for the comparison group should be explained more explicitly. | As in general, timing of treatment is not acknowledged in effectiveness analysis we understand that the sampling scheme is hard to grasp. Details (including figures) on the sampling is provided in Jonéus et al. (2021). However, we understand that the writing was to scant. We now included more than half a page where we provided the intuition for the sampling procedure (see lines 117-146). |
| 3 | The authors should provide a more detailed explanation of the Elixhouser comorbidity index and its relevance to the study. Additionally, including references for the mentioned studies would be helpful. | The present version provides a reference to (Lieffers et al., 2011) who compares Charlson and Elixhauser comorbidity measures to predict colorectal cancer survival using administrative health data (see lines 155-158). They state that the Elixhauser index is a superior comorbidity risk-adjustment method. |
|  | Results: The results section is well-structured and presents the findings clearly. However, there are a few points that could be addressed for further clarity. Specifically: | |
| 1 | The analysis of skeleton-related events (SRE) and severe pain reveals interesting findings. The higher proportion of SRE in the NHT group suggests a potential association with the treatment. However, due to the higher mortality in the NHT group, the estimation of effects requires bounds to account for observation bias. The authors adequately address this limitation. | We do not find any critique in this paragraph, why we did not make any revision. |
| 2 | The analysis of early and late prescriptions of NHT indicates a potential difference in the effects depending on the timing of prescription. However, the overlapping confidence intervals and the lack of a clear pattern prevent firm conclusions. This finding highlights the need for further investigation and does not allow for strong generalizations. | As we do not make any strong generalization, we do not find any critique in this paragraph. For that reason, we did not make any revision. |
| 3 | The discussion of missing data and the assessment of missingness are crucial for understanding the limitations of the study. By treating missing observations as missing at random, the authors address this issue appropriately. However, the impact of missing data on the results should be acknowledged and discussed further. | We added two sentences (see lines 350-352) as disclaimer for the possibility that the missing data may be a problem for the sensitivity analysis. |
| 4 | The analysis of potential confounders, such as age and metastases, provides insights into the robustness of the mortality estimates. The derived lower limits and comparisons with known covariates help assess the potential for reversed effects. The authors appropriately discuss the limitations and implications of these findings | We do not find any critique in this paragraph, why we did not make any revision. |
| 5 | The article would benefit from providing a summary of the main findings at the end of the results section. This would allow readers to quickly grasp the key outcomes without having to revisit the entire section. | We have done so (see lines 322-328). |
|  | Discussion: Generally, discussion section of the article provides a comprehensive analysis of the main results and their implications. However, there are a few areas where additional clarification and expansion would be beneficial. Below are specific comments and suggestions for improvement. | |
| 1 | In paragraph 1, it would be helpful to provide more context regarding the study by Johansson et al. (2021) and its relevance to the current research. | Johansson et al. (2021) is the pre-analysis plan for the present study. As a part or the protocol we conducted a qualitative study on doctors prescribing the drugs examined in this study. This is clarified in the present version of the paper (see lines 428-430) |
| 2 | The authors discuss the potential reasons behind the higher mortality in the NHT group, mentioning the lack of clinical trial evidence and concerns regarding the potential long-term side effects of NHT. Expanding on these potential reasons and discussing alternative hypotheses would contribute to the understanding of the findings. | We do not know how to address this comment. The reason is that we do not have this discussion in the Discussion section nor in the rest of the document. We also would like to avoid speculation about possible reasons for the mortality difference. The clinicians with whom we conducted the qualitative yet unpublished research emphasize that Docetaxel is more effective than NHT, but the tolerance is worse. These opinions need to be verified in future comparative effectiveness evaluations in which not only effectiveness differences is captured but also side-effect differences. |
| 3 | The authors mention the strengths of the study, such as the large sample size and the use of registry data. However, it would be beneficial to provide a more comprehensive overview of the study's strengths and limitations in a dedicated paragraph. | We struggled with this comment. The reason is that we do have a discussion on the strength of the study in this section. However, we added a paragraph on strengths and weakness, by combing comment 3 and 4, at the end in the Discussion section (see lines 449-463) |
| 4 | It would be beneficial to include a brief discussion of the implications of the results for clinical practice or future research. Additionally, highlighting the main limitations and potential areas for further investigation would contribute to the completeness. | We have added a paragraph at the end in the Discussion section (see lines 449-463). |
|  | It would be beneficial to include a brief discussion of the implications of the results for clinical practice or future research. Additionally, highlighting the main limitations and potential areas for further investigation would contribute to the completeness. | |
