# Supplementary material for: Novel hormonal therapy versus standard of care—A registry-based comparative effectiveness evaluation for mCRPC-patients
Source: PLoS One. 2024 Feb 14;19(2):e0290833. doi: 10.1371/journal.pone.0290833 (PMC10866493; doi:10.1371/journal.pone.0290833)
Supplement: Attachment — Submitted filename: Hormonal therapy Reviewer comments.docx [file pone.0290833.s017.docx]

**Review Comments: Novel hormonal therapy versus Standard of Care -- a registry2 based comparative effectiveness evaluation for mCRPC-patients**

Generally, the manuscript titled "Novel hormonal therapy versus Standard of Care - a registry-based comparative effectiveness evaluation for mCRPC patients" provides valuable insights into the comparative effectiveness of Novel Hormonal Therapy (NHT) versus Standard of Care (SoC) for patients with metastatic castration-resistant prostate cancer (mCRPC). The study objectives and methodology are generally well-described, but there are several areas that require further clarification and improvement. Below, I provide specific comments and suggestions for each section of the manuscript.

**Abstract**:

The abstract provides a clear understanding of the study objectives and methodology. there are a few areas that could benefit from further clarification and improvement. Specifically:

Line 14: The statement "one of the few comparative effectiveness evaluations" needs to be supported by evidence. It would be helpful to mention any existing studies that have evaluated the comparative effectiveness of NHT and SoC in mCRPC.

Line 28: The abstract states that there is a substantial increase in mortality for patients prescribed with NHT, but it would be helpful to quantify this increase and provide a measure of effect size or a confidence interval.

Line 32: The statement "We can also rule out a positive effect on skeleton-related events (SRE) for the NHT patients" should be supported by specific findings or statistical results.

**Introduction:**

The introduction provides an overview of the study objectives and methodology. However, there are a few areas that could benefit from further clarification and improvement. Specifically:

Line 47: The statement that "there is only one study comparing chemotherapy and NHT" should be supported by a citation to validate this claim.

Line 49: The authors mention differences in baseline characteristics between patients on docetaxel and NHT but do not clearly state whether these differences were adjusted for in their analyses. This should be clarified.

**Methods:**

The methods section is well-structured and provides a comprehensive overview of the research design. However, there are a few areas that require clarification and improvement. Specifically:

1. The authors should consider expanding the methods section to provide more context and justification for the chosen entropy balancing methodology. This would enhance the understanding of readers who may not be familiar with the statistical techniques.
2. The rationale behind the selection of a 36-month window for NHT-treated patients and the choice of June 2008 to June 2010 for the comparison group should be explained more explicitly.
3. The authors should provide a more detailed explanation of the Elixhouser comorbidity index and its relevance to the study. Additionally, including references for the mentioned studies would be helpful.

**Results:**

The results section is well-structured and presents the findings clearly. However, there are a few points that could be addressed for further clarity. Specifically:

1. The analysis of skeleton-related events (SRE) and severe pain reveals interesting findings. The higher proportion of SRE in the NHT group suggests a potential association with the treatment. However, due to the higher mortality in the NHT group, the estimation of effects requires bounds to account for observation bias. The authors adequately address this limitation.
2. The analysis of early and late prescriptions of NHT indicates a potential difference in the effects depending on the timing of prescription. However, the overlapping confidence intervals and the lack of a clear pattern prevent firm conclusions. This finding highlights the need for further investigation and does not allow for strong generalizations.
3. The discussion of missing data and the assessment of missingness are crucial for understanding the limitations of the study. By treating missing observations as missing at random, the authors address this issue appropriately. However, the impact of missing data on the results should be acknowledged and discussed further.
4. The analysis of potential confounders, such as age and metastases, provides insights into the robustness of the mortality estimates. The derived lower limits and comparisons with known covariates help assess the potential for reversed effects. The authors appropriately discuss the limitations and implications of these findings.
5. The article would benefit from providing a summary of the main findings at the end of the results section. This would allow readers to quickly grasp the key outcomes without having to revisit the entire section.

**Discussion:**

Generally, discussion section of the article provides a comprehensive analysis of the main results and their implications. However, there are a few areas where additional clarification and expansion would be beneficial. Below are specific comments and suggestions for improvement.

1. In paragraph 1, it would be helpful to provide more context regarding the study by Johansson et al. (2021) and its relevance to the current research.
2. The authors discuss the potential reasons behind the higher mortality in the NHT group, mentioning the lack of clinical trial evidence and concerns regarding the potential long-term side effects of NHT. Expanding on these potential reasons and discussing alternative hypotheses would contribute to the understanding of the findings.
3. The authors mention the strengths of the study, such as the large sample size and the use of registry data. However, it would be beneficial to provide a more comprehensive overview of the study's strengths and limitations in a dedicated paragraph.
4. It would be beneficial to include a brief discussion of the implications of the results for clinical practice or future research. Additionally, highlighting the main limitations and potential areas for further investigation would contribute to the completeness.

Generally, this study provides valuable insights into the comparative effectiveness of NHT versus SoC for mCRPC patients. Addressing the points mentioned above will significantly strengthen the manuscript and enhance its contribution to the field.
